# Microglia modulate hippocampal synaptic transmission and sleep duration along the light/dark cycle

**DOI:** 10.1002/glia.24090

**Published:** 2021-09-06

**Authors:** Giorgio Corsi, Katherine Picard, Maria Amalia di Castro, Stefano Garofalo, Federico Tucci, Giuseppina Chece, Claudio del Percio, Maria Teresa Golia, Marcello Raspa, Ferdinando Scavizzi, Fanny Decoeur, Clotilde Lauro, Mara Rigamonti, Fabio Iannello, Davide Antonio Ragozzino, Eleonora Russo, Giovanni Bernardini, Agnès Nadjar, Marie Eve Tremblay, Claudio Babiloni, Laura Maggi, Cristina Limatola

**Affiliations:** ^1^ Department of Physiology and Pharmacology Sapienza University of Rome Rome Italy; ^2^ Axe Neurosciences, Centre de Recherche du CHU de Québec Université Laval Quebec City Quebec Canada; ^3^ Department of Neurology San Raffaele of Cassino Cassino (FR) Italy; ^4^ National Research Council, Institute of Biochemistry and Cell Biology (EMMA/Infrafrontier/IMPC International Campus “A. Buzzati‐Traverso” Rome Italy; ^5^ INRAE, Bordeaux INP, NutriNeuro UMR 1286 Bordeaux University Bordeaux France; ^6^ Tecniplast S.P.A. Buguggiate Varese Italy; ^7^ Department of Molecular Medicine Sapienza University Rome Italy; ^8^ INSERM, Neurocentre Magendie, Physiopathologie de la Plasticité Neuronale Bordeaux France; ^9^ Division of Medical Sciences University of Victoria Victoria British Columbia Canada; ^10^ The Department of Biochemistry and Molecular Biology The University of British Columbia Vancouver British Columbia Canada; ^11^ Department of Physiology and Pharmacology Sapienza University, Laboratory affiliated to Istituto Pasteur Italia Rome Italy; ^12^ Department of Neurophysiology, Neuropharmacology, Inflammaging IRCCS Neuromed Pozzilli Italy

**Keywords:** cx3cr1, electroencephalography, long‐term potentiation, microglial depletion, miniature excitatory post‐synaptic currents, sleep, spontaneous excitatory post‐synaptic currents

## Abstract

Microglia, the brain's resident macrophages, actively contribute to the homeostasis of cerebral parenchyma by sensing neuronal activity and supporting synaptic remodeling and plasticity. While several studies demonstrated different roles for astrocytes in sleep, the contribution of microglia in the regulation of sleep/wake cycle and in the modulation of synaptic activity in the different day phases has not been deeply investigated. Using light as a zeitgeber cue, we studied the effects of microglial depletion with the colony stimulating factor‐1 receptor antagonist PLX5622 on the sleep/wake cycle and on hippocampal synaptic transmission in male mice. Our data demonstrate that almost complete microglial depletion increases the duration of NREM sleep and reduces the hippocampal excitatory neurotransmission. The fractalkine receptor CX3CR1 plays a relevant role in these effects, because *cx3cr1* ^*GFP/GFP*^ mice recapitulate what found in PLX5622‐treated mice. Furthermore, during the light phase, microglia express lower levels of *cx3cr1* and a reduction of *cx3cr1* expression is also observed when cultured microglial cells are stimulated by ATP, a purinergic molecule released during sleep. Our findings suggest that microglia participate in the regulation of sleep, adapting their *cx3cr1* expression in response to the light/dark phase, and modulating synaptic activity in a phase‐dependent manner.

## Main Points

- Neuronal spontaneous activity and microglial CX3CR1 increase in the dark.
- Microglia alterations affect sleep and synaptic transmission in a phase‐dependent manner.
- CX3CR1 mediates microglial effects on neuronal activity in the light/dark cycle.

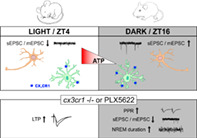

## 1 INTRODUCTION

The environmental light changes caused by the 24‐h period of Earth's rotation around its axis modulate the internal clock in our brain, triggering cycles of alertness and sleepiness in the circadian rhythm. The role of glial cells in the modulation of the biological circadian clock is a matter of intense research (Ingiosi et al., 2013; Garofalo et al., 2020). Several studies point to astrocytes as major determinants of the glial contribution to synaptic homeostasis during the sleep/wake cycle, through the IP_3_/Ca^2+^ signaling pathway (Foley et al., 2017), modulating the extracellular levels of ATP and adenosine (Halassa et al., 2009; Nadjar et al., 2013; Porkka‐Heiskanen et al., 1997) and participating to the glymphatic system for the clearance of metabolites and chemicals from the extracellular space of the brain (Xie et al., 2013). However, key roles have been hypothesized also for microglia during normal sleep and upon sleep disturbances (Bellesi et al., 2017; Choudhury et al., 2020) as well as in neural plasticity processes associated with synaptic remodeling during sleep (Yang et al., 2014; Niethard et al., 2017; de Vivo et al., 2017; Tuan and Lee, 2019; Stanhope et al., 2020; Zhou et al., 2020). In particular, microglial cells undergo molecular alterations along the sleep and wake phases, expressing different patterns of cytokines upon inflammatory challenge (Fonken et al., 2015). Microglia are modulated by the arousal state (Stowell et al., 2019; Liu et al., 2019) and by sleep deprivation, with effects on their expression of receptors for neurotransmitters, hormones and cytokines involved in regulating process motility and phagocytic activity (Wisor et al., 2011). Most studies performed to investigate the effect of sleep and wake on synapses and glial cells used models of sleep deprivation where the wake phase differs for the level and quality of stimuli used to avoid sleep (Havekes and Aton, 2020). To limit possible confounding interference induced by animal manipulation, we used light as a zeitgeber cue, exposing mice to 12:12 light/dark cycles (light: Zeitgeber Time [ZT] 0‐ZT12, dark: ZT12‐ZT0). We investigated the role of microglial cells by depletion studies, taking advantage of the role exerted by colony stimulating factor‐1 receptor (CSF‐1R) signaling on myeloid lineage cells (Ginhoux et al., 2010) and of previous reports demonstrating that CSF‐1R deletion or inhibition leads to a near complete elimination of microglial cells (Erblich et al., 2011, Elmore et al., 2014). We treated mice with PLX5622, a CSF‐1R inhibitor that drastically reduces the density of microglial cells in different brain regions, and we analyzed different endpoints: (1) the time spent in sleep or active states during the light and dark phases of the day; (2) the basal excitatory synaptic transmission; and (3) neuronal plasticity processes in the hippocampal region at ZT4 (light) and ZT16 (dark). These two time points were selected for being far from the light changes and thus better representing constant behavioral conditions. We described that, in the nearly complete absence of microglia, mice spent more time sleeping during the dark phase. The depletion of microglia also affected excitatory synaptic transmission in a phase‐dependent way: (1) abolishing the differences in spontaneous excitatory postsynaptic currents (sEPSC) and miniature EPSC (mEPSC) amplitude and sEPSC frequency between the dark and light phases and (2) increasing hippocampal long‐term potentiation (LTP) exclusively during the light phase.

The chemokine fractalkine (CX3CL1) receptor (CX3CR1) is highly expressed in microglia and its genetic deletion in mice disclosed multiple regulatory roles of CX3CR1 on brain development, synapsis maturation, and neuronal function (Paolicelli et al., 2011; Bolós et al., 2017), as well as on neuronal survival in models on neuropathology (Limatola and Ransohoff, 2014). To investigate whether the effects induced by microglial depletion were at least in part dependent on microglial CX3CR1 signaling, the sleep/wake cycle and the excitatory synaptic transmission along the light/dark cycle were also investigated in *cx3cr1* ^*GFP/GFP*^ mice, where CX3CR1 is deleted (Jung et al., 2000), with outcomes comparable to microglia‐depleted mice. Furthermore, we report that microglial *cx3cr1* expression is lower during the light phase and that, in vitro, it is modulated by the sleep‐dependent metabolite ATP, thus suggesting that CX3CR1 plays a central role in regulating microglial responses during the light/dark cycle.

In conclusion, we demonstrated that under physiological conditions, microglia affect the duration of sleep and are necessary for synaptic changes occurring during the wake phase, disclosing also a key role for CX3CR1 in sleep–wake cycle.

## 2 MATERIALS AND METHODS

### 2.1 Animals

Experiments described in the present work were approved by the Italian Ministry of Health in accordance with the guidelines on the ethical use of animals from the European Community Council Directive of September 22, 2010 (2010/63/EU), the Italian D. Leg. 26/2014. All possible efforts were made to minimize animal suffering and to reduce the number of animals used per condition by calculating the necessary sample size before performing the experiments.

To avoid sex‐ and gonadal hormone‐related variations, we decided to always use male mice. Eight‐ to 10‐week‐old C57BL/6N mice were obtained from European Mouse Mutant Archive (EMMA, Monterotondo, Italy); homozygous *cx3cr1* ^*GFP/GFP*^ mice Cat# JAX:005582, RRID: IMSR_JAX:005582 (https://www.jax.org/strain/005582) and C57BL6/J mice Cat# JAX:000664, RRID: IMSR_JAX:000664 (https://www.jax.org/strain/000664) were obtained from The Jackson Laboratory (Bar Harbor, ME, USA). C57BL6/J mice were used as controls for *cx3cr1* ^*GFP/GFP*^ mice, given the similarity in the genetic background, as suggested by The Jackson Laboratory. For real‐time PCR analyses and electrophysiological recordings, mice were rapidly anesthetized inside the home cage.

### 2.2 Housing and light conditions

Mice were housed in standard breeding cages at a constant temperature (22 ± 1°C) and relative humidity (50%). Food and water were available ad libitum.

Before any experimental intervention, all mice were housed for at least 7 days upon arrival with a 12:12 h light/dark cycle (light 7:00, ZT0; dark 19:00, ZT12). The same light conditions were kept for all experimental phases, except for the electrophysiology experiments performed in hippocampal slices during the dark phase (see below).

### 2.3 PLX5622 treatment

Microglial depletion was achieved by administering the Plexxikon CSF1R inhibitor PLX5622 (1200 PPM added to AIN‐76A chow, Research Diets) to C57BL/6N mice at least 7 days before experiments and its administration continued until the end of the experiments. Age‐matched control group received control diet (AIN‐76A, Research Diets).

### 2.4 Video‐electroencephalography and electromyography recordings

We performed 24 h electroencephalography (EEG) recordings in mice treated for 2 weeks with PLX5622 (*n* = 9), in *cx3cr1* ^*GFP/GFP*^ mice (*n* = 7) and in age‐matched control mice (C57BL/6N, *n* = 10; C57BL/6J, n = 4). Experimental procedures were validated in European project “PharmaCog” and reported in detail elsewhere (Del Percio et al., 2017). Before surgery, mice were acclimatized to the recording cages for 7 days (2–5 min per day). Stainless steel insulated epidural EEG exploring electrodes (Bilaney Consultants GmbH, Cat# E363/20/SPC) were implanted under isoflurane (3%) anesthesia and intraperitoneal injection of Rompun (20 mg/ml) at 75 mg/kg + Zoletil (50 mg/ml) at 20 mg/kg over the anterior frontal (from Paxinos atlas Bregma: AP +2.8 mm and ML at −0.5 mm) and posterior parietal (AP −2.0 mm and ML −2.0 mm) cortical regions. They were referred to an electrode placed in the cerebellum (nose bone ground). Another electrode (15 mm shaft, Bilaney Consultants GmbH, Cat# E363‐3‐SPC) was positioned into the dorsal back muscles to record electromyographic (EMG) activity useful to monitor the motor activity. A few hours after the surgery, mice were treated by systemic analgesics and antibiotics. PLX5622 and control chow administration started at this moment and continued until the end of the experiment. After 1 week of full recovery following the electrodes implantation, mice experienced 1 week of handling and plugging–unplugging operations every day (2–5 min per day). The day before the experiment, the mice were placed in the recording cage for acclimatization.

Grass Technologies ©2011 (Twin software 4.5.3.23) was used for simultaneous EEG, EMG, and video recordings performed on pairs of mice for 24 h (12 h lights and 12 h dark). Each pair was formed by one PLX5622 or *cx3cr1* ^*GFP/GFP*^ and one control mouse. The mice were simultaneously connected to a system consisting of AS40 Amplifier by a flexible cable. The EEG and EMG data were collected with a sampling rate of 200 Hz and an antialiasing band pass analogue filter.

The video‐EEG–EMG analysis was initially performed in five controls and five PLX5622‐treated mice for the whole dark and light period, only excluding the first hour after light change (22 h in total). The results of this exploratory analysis are reported in the supplementary results (Figure S2) and revealed that comparable information can be obtained by analyzing shorter periods (7 h each phase). In consideration of the time‐consuming procedure of analysis, the EEG–EMG analysis was limited from ZT4 to ZT11 in the light period and from ZT13 to ZT20 in the dark period.

Each video and EEG–EMG recording epoch lasting 8 s has been classified into the following behavioral classes:

1. Active behavior (movement condition). The mice performed overt movements in the cage for most of the given epoch of 8 s. The movements were characterized by ample displacements of body parts such as trunk, head, and forelimbs. Significant EMG activity was expected in this behavioral condition.
2. Passive behavior (passive wake condition). The mice showed no movement (i.e., substantial immobility) periods intermingled with small movements of the trunk, head, and forelimbs for the majority of 8 s. The maximal duration of immobility considering two contiguous epochs was 8 s. This criterion was expected to minimize the risk that “passive condition” could be misclassified as sleep and vice–versa.
3. Immobility condition. The mice performed no movement of the trunk, head, and forelimbs for at least 20 s across three or more epochs. This condition was associated with a low EMG activity and required the analysis of the ongoing EEG and EMG waves for a discrimination between a condition of passive behavior and sleep (see below).
4. Undefined. Each epoch classified as undefined showed a mix of behavioral classes or lack of clarity about the behavioral situation of the mice. The number of undefined epochs in all groups of mice was less that 1 out of 1000 epochs, and there were no statistical differences in the undefined epochs among the groups (Mann Whitney *U* test, *p* > .05 uncorrected). Since the undefined epochs were negligible, they were not considered in the calculation of the percentage of time spent in sleep and wake. Consequently, the summed percentage of time spent in sleep and wake was equal to 100% in the reported results due to that approximation.

Afterward, the periods classified as “Immobility” were evaluated based on a standard visual analysis of EEG and EMG data. The classification of the animal immobility as non‐rapid eye movement (NREM) sleep was based on the observation of sleep spindles (quite infrequent) and EEG slow sleep waves, whereas the classification as rapid eye movement (REM) sleep was based on the behavioral sleep state associated with low EMG activity and the following EEG features: dominant theta waves and no sleep spindles and EEG slow waves (Del Percio et al., 2017).

### 2.5 Slice preparation for electrophysiology

For electrophysiology, mice were sacrificed during the light phase at ZT4 and during the dark phase at ZT16. In order to perform all the electrophysiological recordings during the working time of the day, mice sacrificed at ZT16 received a light/dark cycle inversion (light on at 19:00) for at least 14 days prior to start the control chow or PLX5622 treatment, for microglia depletion in wild‐type mice. In this way, slices were harvested at 11:00 for all groups. To verify that light/dark cycle inversion did not affect the results of the experiments, a group of control mice receiving the normal light/dark cycle was sacrificed at 23:00. No significant differences were observed between the ZT16 groups in normal and inverted cycles and data were pooled.

For slicing preparation, anesthetized animals were decapitated and the whole brains were rapidly removed from the skull and immersed for 10 min in ice‐cold artificial cerebrospinal fluid (ACSF; composition in mM: NaCl 125, KCl 4, CaCl_2_ 2.5, MgSO_4_ 1.5, NaH_2_PO_4_ 1, NaHCO_3_ 26, and glucose 10; 295–300 mOsm), continuously oxygenated with 95% O_2_ and 5% CO_2_ to maintain the proper pH (7.4). Transverse 350 μm slices were cut at 4°C with a vibratome (Thermo Scientific, USA) and then placed in a chamber containing oxygenated ACSF. After their preparation, slices were allowed to recover for at least 1 h at 30°C.

### 2.6 Patch‐clamp recordings

Hippocampal slices for patch clamp recordings were prepared from C57BL/6N (*n* = 12), PLX5622‐treated mice (*n* = 9), C57BL6J (*n* = 10) and *cx3cr1* ^*GFP/GFP*^ (*n* = 9). Whole‐cell patch clamp recordings were performed on CA1 pyramidal neurons at room temperature by using a Multiclamp 700B amplifier (Molecular Devices, USA). The ACSF (composition in mM: NaCl 125, KCl 2, CaCl_2_ 2, MgCl_2_ 1.2, NaH_2_PO_4_ 1.2, NaHCO_3_ 25, and glucose 10) was perfused at a rate of approximately 2 ml/min by using a gravity‐driven perfusion system. Glass electrodes (3–4 MΩ) were pulled with a vertical puller (PC‐10, Narishige). Pipette were filled with 135 mM Cs Methanesulfonate, 10 mM Hepes, 0.5 mM EGTA, 2 mM Mg‐ATP, 0.3 mM Na_3_‐GTP, and 2 mM MgCl_2_ (295–300 mOsm, pH 7.2). Cell capacitance was constantly monitored over the time and experiments where access resistance changed more than 20% were discarded.

Spontaneous excitatory post‐synaptic currents (sEPSCs) were recorded in ACSF by holding the cell at the reversal potential of GABA current (−70 mV). Miniature excitatory post‐synaptic currents (mEPSCs) were isolated by adding Tetrodotoxin (0.5 mM, TOCRIS) to ACSF in the perfusion line for at least 10 min before starting acquisition. Signals were acquired (sampling 10 kHz, low‐pass filtered 2 kHz) with DigiData‐1440A using pCLAMP‐v10 software (Molecular Devices, USA).

Analysis of sEPSCs and mEPSCs was performed offline using MiniAnalysis software (Mini Analysis, Synaptosoft Fort Lee, NJ, USA) with the threshold for detection set at 5 pA. Cumulative probability curves were constructed by considering 100 consecutive events each cell.

### 2.7 Field excitatory post synaptic potential recordings

Hippocampal slices for field recordings were prepared from C57BL/6N (n = 25), PLX5622‐treated mice (*n* = 14), C57BL6J (*n* = 9) and *cx3cr1* ^*GFP/GFP*^ (*n* = 11).

Slices were transferred to the slice‐recording chamber interface (BSC1, Scientific System Design Inc), maintained at 30–32°C and constantly superfused at the rate of 2.5 ml/min with oxygenated ACSF. At the beginning of each recording, a concentric bipolar stimulating electrode (SNE‐100X 50 mm long Elektronik–Harvard Apparatus GmbH) was placed in the hippocampus CA1 stratum radiatum for stimulation of Shaffer collateral pathway projection to CA1. Stimuli consisted of 100 μs constant current pulses of variable intensities, applied at 0.05 Hz. A glass micropipette (0.5–1 MΩ) filled with ACSF was placed in the CA1 region, at 200–600 μm from the stimulating electrode, in order to measure orthodromically evoked fEPSP. The paired‐pulse ratio (PPR) was measured from responses to two synaptic stimuli at 50 ms interstimulus interval. PPR was calculated as the ratio between the field excitatory post synaptic potential (fEPSP) amplitude evoked by the second stimulus (A2) and that by the first (A1; A2/A1). Only the slices that showed stable fEPSP amplitudes were included in the experiments. Field EPSP was recorded and filtered (low pass at 1 kHz) with an Axopatch 200A amplifier (Axon Instruments, CA) and digitized at 10 kHz with an A/D converter (Digidata 1322A, Axon Instruments). Data acquisition was stored on a computer using pClamp 9 software and analyzed offline with Clampfit 10 software (both from Axon Instruments).

### 2.8 Isolation of CD11b ^+^ cells from adult mice from brain areas

C57BL6/N mice (ZT4 *n* = 15, ZT16 *n* = 13) were taken from cages and rapidly anesthetized, in no more than 1 min. Animals were intracardially perfused with PBS, brains were extracted, and the hippocampus, prefrontal cortex, and hypothalamus were isolated and grinded in Hank's Balanced Salt Solution (ThermoFisher) to obtain single‐cell suspension. Cells were immediately processed for MACS MicroBeads (Miltenyi) separation. CD11b Microbeads (Miltenyi Biotec, Cat# 130‐049‐601; https://www.miltenyibiotec.com/IT-en/products/cd11b-microbeads-human-and-mouse.html#130-049-601) were used to magnetically label CD11b^+^ cells. The cell suspension was loaded onto a MACS column placed in the magnetic field of a MACS Separator and the negative fraction was collected. After removing the magnetic field, CD11b^+^ cells were eluted as a positive fraction and the purity was 99% (Garofalo et al., 2017).

### 2.9 ATP treatment of microglial primary cultures from pups

Microglial cells were obtained from mixed glia cultures derived from the cerebral cortices of post‐natal day 0–2 (P0–P2) C57BL6/J mice. Cortices were chopped and digested in 15 U/ml papain (Sigma, Cat# P3125) for 20 min at 37°C. Cell suspensions were plated (5 × 10^5^ cells/cm^2^) on poly‐L‐lysine hydrobromide (Sigma, Cat# P2636) (0.1 mg/ml) coated flasks in growth medium supplemented with 10% FBS. After 9–11 days, cultures were shaken for 2 h at 37°C to detach and collect microglia cells. These procedures gave almost pure microglial cell populations as previously described (Lauro et al., 2010). Cells were seeded on poly‐L‐lysine coated 12 well plates (40 × 10^4^ cells) and 2 days after they were treated with lipopolysaccharide (LPS, 100 ng/ml) (Sigma Cat# L4391) or ATP (Sigma, Cat# A6419) (1 mM, 100, 50, and 10 μM) for 4 h.

### 2.10 ATP treatment of microglial primary cultures from adult mice

Sixty‐day‐old mice were deeply anesthetized and intracardially perfused with ice cold PBS. Brains were removed, each hemisphere was cut into small pieces and disrupted in a glass‐teflon homogenizer. Cell suspension was applied to a 30‐μm cell strainer, labeled with CD11b^+^ Microbeads and passed through MACS Columns (Miltenyi Biotec). These procedures gave almost pure microglial cell populations as previously described (Garofalo et al., 2017). Microglia cells were plated in DMEM/F12 plus GM‐CSF (5 ng/ml) on poly‐L‐lysine coated 24‐well plate (20 × 10^4^ cells). After 4 days, cells were treated with ATP 100 μM for 4 h.

### 2.11 Analysis of cx3cr1 mRNA expression

CD11b+ cells isolated from tissue and cultured microglial cells were lysed in Trizol reagent (Invitrogen) for isolation of total RNA and purified by isopropanol precipitation; quality and yield were verified using the NANODROP One system (Thermo Scientific Scientific).

For RT‐qPCR, reverse transcription reaction was performed in a thermocycler using IScript TM RT Supermix (Biorad) under the following conditions: incubation, 25°C, 5′; reverse transcription, 42°C, 45′; inactivation, 85°C, 5′. RT‐qPCR was carried out in a I‐Cycler IQ Multicolor RT‐PCR Detection System using SSO Fast Eva Green Supermix (Biorad). The PCR protocol consisted of 40 cycles at 95°C, 30″ and 60°C, 30″. For quantification analysis, the comparative Threshold Cycle (Ct) method was used. The Ct values from each gene were normalized to the Ct value of *gapdh* in the same cDNA samples. Relative quantification was performed using the 2^−ΔΔCt^ method (Schmittgen and Livak, 2008) and expressed as fold increase in arbitrary values.

Primer sequences (5′–3′): *gapdh*, forward (f): TCGTCCCGTAGACAAAATGG, reverse (r): TTGAGGTCAATGAAGGGGTC; *cx3cr1*, f: TGACTGGCACTTCCTG‐CAGA, r: AGGGCGTAGAAGACGGACAG.

### 2.12 Immunofluorescence and FACS analysis

For in vitro cell culture studies, primary microglial cells obtained from C57BL/6N pups (P0‐2) were treated with vehicle, LPS (100 ng/ml) and ATP (100 μM and 1 mM) for 4 h. After incubation, cells were detached from plates using dispase (1 mg/ml), washed and stained with anti‐CX3CR1 specific monoclonal antibody (PerCP Fluorochrome, Biolegend SA011F11 149009, dilution 1/60). Cells were analyzed by flow cytometry using a FACSCanto II (BD Biosciences), and data were elaborated using FlowJo v10.7. The level of CX3CR1 expression (Median Fluorescence Intensity: MFI) was quantified after subtracting control staining (Fluorescence Minus One: FMO).

### 2.13 Statistical analyses

Statistical significance was assessed by Student's *t*‐test, one‐way or two‐way ANOVA for parametrical data, as indicated. In particular, for PCR, statistical analyses were conducted using one‐way ANOVA followed by Dunn's and Tukey post hoc test (Sigmaplot software). Electrophysiological experiments were analyzed by two‐way ANOVAs, considering subject (controls, PLX5622 and *cx3cr1* ^*GFP/GFP*^ mice) and environmental condition (ZT4 and ZT16) as the between‐subject variables (Sigmaplot software). Post hoc comparisons were performed using Holm–Sidak test. For the cumulative distribution analyses, we applied the Kolmogorov–Smirnov two‐sample test (KS) using the MiniAnalysis software (Synaptosoft Fort Lee, NJ, USA). All mean differences were considered statistically significant when *p* < .05.

For EEG experiments, a statistical analysis of the epochs lasting 8 s data was performed by Statistica 10.0 packages (StatSoft Inc., www.statsoft.com). The primary analysis of those epochs was performed on the sleep duration, whereas the secondary analysis was focused on the duration of the exploratory movements (the latter was quantitatively tested in parallel experiments using the DVC cages). Due to the sample size, non‐parametric tests were used. The Wilcoxon test was used for a within‐group analysis of the sleep (NREM + REM sleep) and movement duration between the light and the dark periods. The Mann–Whitney *U* test was used for the between‐group analysis.

For the primary analysis of REM and NREM sleep duration, the *p* value was corrected (Bonferroni) for 8 statistical comparisons, namely *p* < .05/8 = .006 = *p* < .05 corrected. For exploratory purposes, a statistical threshold of *p* < .05 uncorrected was also used. The statistical comparisons were (1) 4 within‐group comparison light vs dark for the C57BL/6, PLX5622, C57BL/6J and *cx3cr1* ^*GFP/GFP*^ groups; (2) 2 between‐group comparisons for the light period for the C57BL/6 versus PLX5622 groups and the C57BL/6J versus *cx3cr1* ^*GFP/GFP*^ groups; and (3) 2 between‐group comparisons for the dark period for the C57BL/6 versus PLX5622 groups and C57BL/6J versus *cx3cr1* ^*GFP/GFP*^ group. Note that almost all sleep periods were spent in the NREM sleep. The same design was used for the secondary analysis of movement durations.

Sample size (*n*/*N*) refers to the number of: (1) slices/mice, for filed cell recording analysis; (2) cells/mice for patch clamp recordings; (3) wells/cultures for in vitro analysis. *N* refers to the number of mice for EEG and motor activity analysis. All data are expressed as mean ± standard error of the mean (SEM).

## 3 RESULTS

### 3.1 Effect of microglial depletion on sleep duration in the light/dark cycle

**FIGURE 1:**
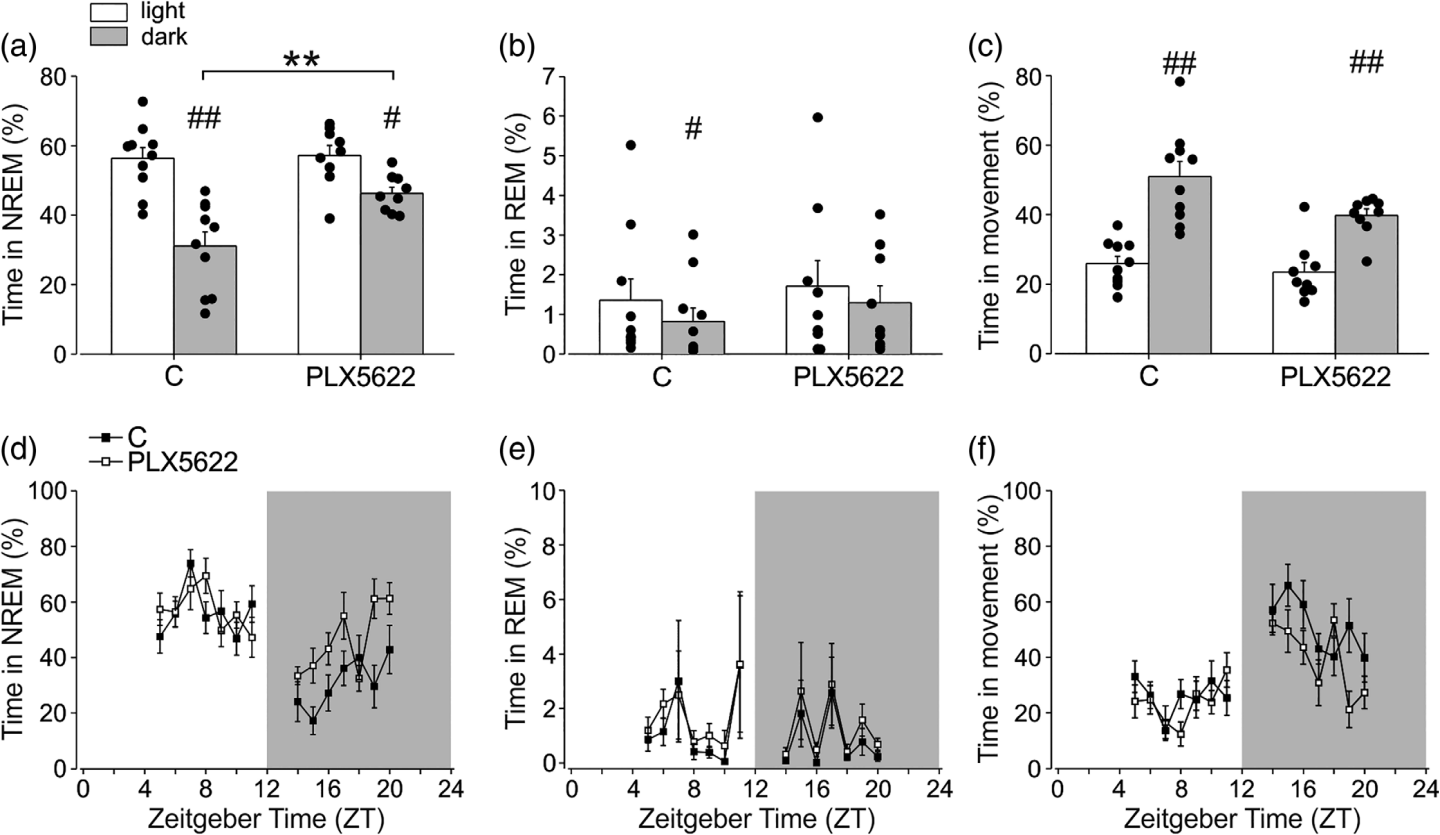
Effect of microglial depletion on sleep and movement duration in the dark and light phases. Mean ± SEM and individual distribution of non‐rapid eye movement (NREM) sleep (a), rapid eye movement (REM) sleep (b) and movement (c) duration in 7 h of light versus 7 h of dark in C57BL/6 (control, C. *n* = 10) and PLX5622‐treated (*n* = 9) mice. The within‐group statistical analysis was performed by Wilcoxon test for paired measures and showed statistical differences for NREM sleep (C ## *p* = .0025 corrected, *z* = 2.80, *t* = 0.00; PLX5622 # *p* = .0054 corrected, *z* = 2.54, *t* = 0.00), REM sleep (C # *p* = .014 uncorrected, *z* = 2.19, *t* = 0.00) and movement duration (C ## *p* = .0025 corrected, *z* = 2.80, *t* = 0.00; PLX5622 ## *p* = .0038 corrected, *z* = 2.66, *t* = 0.00), in light versus dark conditions. The between‐group statistical analysis was performed by Mann–Whitney *U* test for unpaired measures and showed increased NREM sleep in PLX5622‐treated mice compared to C (**p = 0.0031 corrected, *z* = −2.74, *U* = 11.00), (d–f) time‐courses of NREM (d), REM (e) and movement (f) duration at the time‐points analyzed. Data are expressed as a percentage of the total time analyzed and are shown as mean ± SEM

To investigate the effect of microglial cell depletion on the wake–sleep cycle, we treated mice with the CSF‐1R inhibitor PLX5622. This treatment is reported to efficiently and transiently deplete microglia in several brain regions (Huang et al., 2018), and we confirm this result in three different brain regions involved in sleep regulation such as the hypothalamus (the whole structure) (92.81 ± 3.37% depletion), the prefrontal cortex (PFC) (95.28 ± 2.32% depletion) and the hippocampus (93.89 ± 2.76% depletion) (Ono and Yamanaka, 2017; Durán et al., 2018) (Figure S1). Microglia‐depleted mice were analyzed for video, fronto‐parietal EEG and back‐muscle EMG recordings in 24 h and the data analysis was performed for 7 h (from ZT4 to ZT11) in the light phase and 7 h (from ZT13 to ZT20) in the dark phase. As expected, both control and PLX5622 treated mice showed longer sleep (total and NREM sleep) duration during the light phase (Table 1; Figure 1a,d), and longer movement durations during the dark phase (Table 1; Figure 1c,f) and REM sleep was shorter in the dark phase for control mice (Table 1; Figure 1b,d).

**TABLE 1:** Time spent by each group in the different behavioral conditions. Mean ± SEM and percentages of the time (h) spent by each mouse group (i.e., C57BL/6, PLX5622, C57BL/6J, and CX3CR1) in the following behavioral conditions: sleep (non‐rapid eye movement + rapid eyes movement [NREM + REM]), NREM, REM, wakefulness (movement + passive wake), movement, and passive wake durations. The mean values were calculated considering 7 h of the light phase (of the day) and 7 h of the dark phase, which were considered as representative of 24 h based on an exploratory analysis carried out at early stage of the study. In those hours, the 8‐s epochs classified as “undefined” were very few (i.e., < 1 on 1000), so we did not consider them in reporting the percentages of the time spent in sleep and wakefulness. Asterisks in bold indicate the statistically significant differences between the experimental (i.e., PLX5622 or CX3CR1) and control (i.e., C57BL/6 or C57BL/6J) mouse groups (*p* < .05 uncorrected)

		C57BL/6 (*N* = 10)	PLX5622 (*N* = 9)	C57BL/6J (*N* = 4)	CX3CR1 (*N* = 7)
		Mean ± SE (h)	Mean ± SE (h)	Mean ± SE (h)	Mean ± SE (h)
Conditions		% ± SE	% ± SE	% ± SE	% ± SE
Sleep duration (NREM + REM)	Light	4.0 ± 0.2	4.1 ± 0.2	4.4 ± 0.2	4.4 ± 0.3
		57.7% ± 3.0%	58.9% ± 3.2%	62.8% ± 3.1%	62.5% ± 3.9%
	Dark	2.2 ± 0.3	3.3 ± 0.1	2.4 ± 0.2	3.1 ± 0.3
		31.9% ± 4.2%	47.5% (*) ± 1.7%	34.7% ± 2.5%	44.7% (*) ± 4.3%
NREM duration	Light	3.9 ± 0.2	4.0 ± 0.2	4.4 ± 0.2	4.4 ± 0.3
		56.4% ± 3.1%	57.2% ± 2.8%	62.4% ± 3.0%	62.2% ± 3.9%
	Dark	2.2 ± 0.3	3.2 ± 0.1	2.4 ± 0.2	3.1 ± 03
		31.1% ± 4.0%	46.3% (*) ± 1.8%	34.7% ± 2.5%	44.6% (*) ± 4.2%
REM duration	Light	0.1 ± 0.0	0.1 ± 0.0	0.03 ± 0.0	0.02 ± 0.0
		1.4% ± 0.5%	1.7% ± 0.6%	0.4% ± 0.3%	0.3% ± 0.3%
	Dark	0.1 ± 0.0	0.1 ± 0.0	0.003 ± 0.0	0.005 ± 0.0
		0.8% ± 0.3%	1.3% ± 0.4%	0.04% ± 0.04%	0.1% ± 0.1%
Wakefulness duration (movement + passive wake)	Light	3.0 ± 0.2	2.9 ± 0.2	2.6 ± 0.2	2.6 ± 0.3
		42.3% ± 3.0%	41.1% ± 3.2%	37.2% ± 3.1%	37.5% ± 3.9%
	Dark	4.8 ± 0.3	3.7 ± 0.1	4.6 ± 0.2	3.9 ± 0.3
		68.1% ± 4.2%	52.5% (*) ± 1.7%	65.3% ± 2.5%	55.3% (*) ± 4.3%
Movement duration	Light	1.8 ± 0.1	1.6 ± 0.2	2.1 ± 0.3	1.7 ± 0.2
		26.0% ± 2.1%	23.5% ± 2.7%	30.0% ± 3.8%	24.9% ± 2.8%
	Dark	3.6 ± 0.3	2.8 ± 0.1	2.9 ± 0.2	2.9 ± 0.3
		51.0% ± 4.3%	39.8% ± 1.9%	41.1% ± 3.2%	40.9% ± 4.1%
Passive Wake duration	Light	1.1 ± 0.2	1.2 ± 0.1	0.5 h ± 0.1	0.9 ± 0.1
		16.3% ± 2.2%	17.6% ± 2.0%	7.2% ± 1.3%	12.6% (*) ± 1.9%
	Dark	1.2 ± 0.2	0.9 ± 0.1	1.7 ± 0.3	1.0 ± 0.2
		17.1% ± 2.5%	12.7% ± 1.3%	24.2% ± 4.9%	14.4% ± 2.6%

Interestingly, when compared to the control, the PLX5622 group spent a higher amount of time in sleep (total and NREM) during the dark phase (Table 1, Figure 1a,d). Note that mice spent most of the sleep duration in the NREM phase (see Figure 1a,d versus b,e). The latency of the sleep onset was shorter in the PLX5622 group both in light and dark phases (*p* < .05 corrected, Table 1). Detailed results of the analyses within and between groups are reported in Tables S1, S2, and S3.

Similar results for NREM sleep were obtained when EEG data were analyzed for longer periods (11 h in the light phase and 11 h in the dark phase), as shown in Figure S2. All the successive analyses were performed on shorter periods of 7 h.

To better understand the effects of microglial depletion on sleep, we performed analyses on circadian variables after PLX5622 treatment in 12 h light/12 h dark and in 12 h dark/12 h dark cycles. For these analyses, we took advantage of an innovative system, the digital ventilated cages (DVC), that automatically records motor activity for prolonged periods (24 h per day) and with minimal operator intervention, thus avoiding interfering with animal rhythms. Results shown in Figures S3 and S4 show that no overt variations of circadian variables were observed between control and PLX5622 treated mice. Figure S3 shows the comparison of average activity, 24 h activity pattern, acrophase, and diurnality between the control and PLX5622‐treated mice along the light/dark cycle. To study the effect of microglial depletion on circadian time (CT), we investigated the effect of PLX5622 treatment in the dark/dark experimental setting: Figure S4 demonstrates that no significant variation in the average activity, activity onset, and phase shift were observed upon PLX5622 treatment in mice housed in 12 h dark 12 h dark condition, indicating no effects on mouse inner CT.

Analysis of time spent in movement (Figure 1c,f) did not reveal significant differences among control and microglial‐depleted mice, while total wakefulness phase was decreased both in light and dark periods (Table 1). Interestingly, comparable results on NREM sleep duration were obtained using another CSF1R antagonist, PLX3397, that partially depleted microglial cells (around 88%, data not shown) (Jin et al. 2017) with an independent set of analyses (Figure S5), confirming the role of microglia in modulating NREM sleep duration during the dark phase. The REM sleep duration was also not affected by PLX3397 treatment (Figure S5B, E), while the time in the wake phase was also decreased (Figure S5C, F). Note that the duration of REM phase varied in the experimental group shown in Figure 1b and in Supplementary Figure S5B, from about 1% to 6%, possibly due to some experimental differences described in Supplementary Materials.

### 3.2 Microglial depletion affects hippocampal synaptic transmission in a daily‐phase dependent manner

**FIGURE 2:**
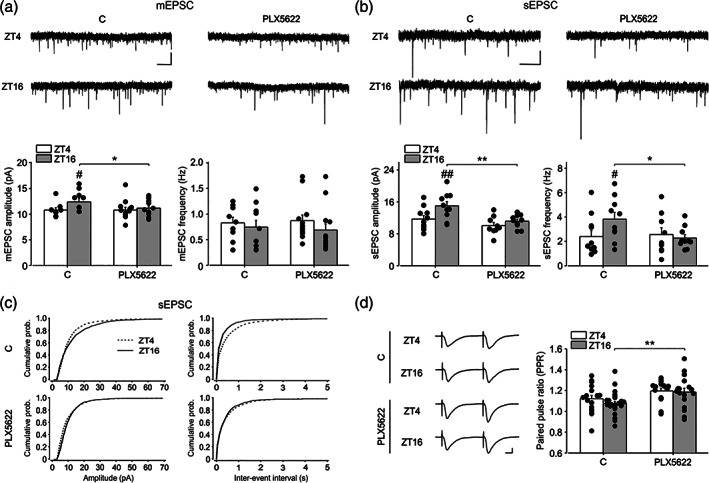
The depletion of microglia alters synaptic transmission across the light–dark cycle. (a) Top: Representative traces of mEPSCs recorded at −70 mV from hippocampal CA1 neurons at ZT4 and ZT16 in control (C) and PLX5622 conditions; scale bars: 2 s (horizontal), 10 pA (vertical). bottom: histograms of the mean amplitude (left) and frequency (right) for mEPSCs at ZT4 and ZT16 in C and in PLX5622 conditions. Miniature EPSC amplitude is increased in C during dark condition (ZT16: 10/4 cells/mice; ZT4: 16/4, *t* = 2.332, *p* = .025) and PLX5622 treatment reduces mEPSC amplitude at ZT16 (ZT16 PLX5622: 13/4 cells/mice, *t* = 2.066, *p* = .045) compared to C, abolishing the difference at the two times considered (ZT4 PLX5622: 13/5, post hoc 13/4, *t* = 0.521, *p* = .605). Frequencies of mEPSC were similar in all groups. (b) Top: representative traces of sEPSC at ZT4 and ZT16 in control (C) and PLX5622 conditions; bottom: histogram of the mean amplitude (left) and frequency (right) for sEPSCs in control conditions (C) and after PLX5622 treatment. Spontaneous EPSC amplitude is increased in C during dark condition (ZT16: 10/4 cells/mice; ZT4: 10/4, *t* = 2.855, *p* = .007) and reduced upon PLX5622 treatment compared to control (ZT16 PLX5622: 10/4 cells/mice, *t* = 3.310, *p* = .002). The frequency of sEPSC is increased in C at ZT16 (*t* = 2.167, *p* = .037) but reduced after PLX5622 treatment only at dark (*t* = 2.292, *p* = .028). (c) Left. The cumulative probability curve for sEPSC amplitude is shifted rightward at ZT16 (*n* = 1200 events) compared to ZT4 (*n* = 900 events) in C (KS, *p* < .001), reflecting increased sEPSC amplitude. Right. The cumulative function for the inter‐event intervals (right) is shifted to the left in control (C) at ZT16 (KS, *p* < .0001), in line with the increased sEPSC frequency. Upon PLX5622 treatment the cumulative functions for both sEPSC amplitude and IEI were similar at the two time points (KS, *p* = .28 and *p* = .39). (d) Representative fEPSP traces (left) and mean values (right) for PPR experiments performed (ISI = 50 ms) at ZT4 and ZT16 in control (17/9 and 31/16 slices/mice) and PLX5622 treated mice (16/7 and 17/7 slices/mice respectively. PPR is increased in PLX5622‐treated mice at ZT16 (*t* = 3.012, *p* = .004). Scale bars: 0.3 mV (vertical), 10 ms (horizontal). Data are shown as mean ± SEM. Statistical analysis was performed with Two‐way ANOVAs, Holm‐Sidak post hoc comparison. *, # *p* < .05, **, ## *p* < .01. Cumulative probability functions were compared with Kolmogorov–Smirnov test

In several brain regions, the number and the morphology of dendritic spines on neurons along with the expression levels of AMPA receptors can change across the light/dark cycle, resulting in an increased frequency and/or amplitude of the mEPSC during the dark phase (Vyazovskiy et al., 2008; Liu et al., 2010; Maret et al., 2011; Yang et al., 2014; Bridi et al., 2020). To investigate whether microglia play a role in the synaptic scaling of miniature events along the light–dark cycle, we performed patch clamp experiments from CA1 hippocampal neurons in control and PLX5622 treated mice, in slices harvested at ZT4 and ZT16. We found that the phase of the day had a main effect on the amplitude of mEPSC (*F*[1,38] = 4.461, *p* = 0.041) with higher values during the dark (ZT16: 12.66 ± 0.54 pA, *n*/*N* = 10/4 cells/mice) relative to the light phase (ZT4: 10.82 ± 0.57 pA, *n*/*N* = 16/4, Holm‐Sidak post hoc analysis *t* = 2.332, *p* = .025) (Figure 2a, left). Microglia depletion reduced the mEPSCs amplitude only at ZT16 (*t* = 2.066, *p* = 0.045) abolishing the difference observed at the two times considered (ZT16: 11.16 ± 0.44 pA *n*/*N* = 13/4; ZT4: 10.82 ± 0.48 *n*/*N* = 13/5, post hoc *t* = 0.521, *p* = 0.605, Figure 2a, left). Conversely, mEPSC frequency was not different in control and PLX 5622‐treated group (ZT16 CTRL: 0.75 ± 0.13 Hz, ZT4 CTRL: 0.83 ± 0.13 Hz, *t* = 0.452, *p* = 0.654; ZT16 PLX: 0.69 ± 0.11 Hz, ZT4 PLX: 0.87 ± 0.11 Hz, *t* = 1.152, *p* = 0.745, Figure 2a, right).

We then measured spontaneous synaptic activity performing the recordings in the absence of TTX, to better approximate the physiological condition. We found a main effect of phase and treatment on the sEPSC amplitude (*F*[1,38] = 6.959, *p* = .012; *F*(1,38) = 11.374, *p* = .002, respectively (Figure 2b, left). Post hoc analysis showed that at ZT16 the mean amplitude of sEPSC was higher (ZT16: 14.91 ± 1.59 pA, *n*/*N* = 10/4 cells; ZT4: 11.68 ± 0.82 pA, *n*/*N* = 10/4 cells, *t* = 2.855, *p* = 0.007, Figure 2b, left) and the cumulative probability curve of sEPSC amplitude was significantly shifted to the right compared to ZT4 group (*p* < 0.001, KS, Figure 2c, left), indicating an increased strength of synaptic transmission in the dark phase. Similar to what observed for mEPSCs, microglia depletion abolished the difference in the mean sEPSC amplitude between ZT4 and ZT16 (ZT4 PLX5622: 9.98 ± 0.86 pA, *n*/*N* = 9/4, *t* = 0.925; *p* = .36) (Figure 2b), resulting in the overlap of the cumulative probability distribution (KS, *p* = .28, Figure 2c, left).

In addition, in ZT16 control group, the frequency of sEPSCs was higher compared to ZT4 (ZT16: 3.702 ± 0.49 Hz, ZT4: 2.2 ± 0.43 Hz, *t* = 2.167, *p* = .037, Figure 2b, right) and the inter‐event interval (IEI) cumulative probability curve shifted toward the left (*p* < .0001, KS, Figure 2c, right) reflecting an increased sEPSC frequency in the dark. PLX5622 treated mice showed similar sEPSC frequency (ZT16 PLX5622 frequency: 2.26 ± 0.48 Hz; ZT4 PLX5622: 2.55 ± 0.51 Hz, *t* = .412, *p* = .683, Figure 2b, right) and cumulative probability curves at the two zeitgeber time considered (*p* = 0.39, KS, Figure 2c, right). We found that both the amplitude and the frequency of sEPSCs were significantly reduced in the ZT16 PLX5622 compared to the ZT16 control group (*t* = 3.310, *p* = .002; and *t* = 2.292, *p* = .028, respectively). Although at ZT4 no statistical difference was observed for the mean values of these parameters between PLX5622 and control group, cumulative distributions for sEPSC amplitudes were shifted to the left for the treated mice indicating a reduction, albeit minimal (*p* < .0001 KS, data not shown). These results indicate that microglial cells have a role in setting the differences in synaptic strength across the light/dark cycle and their removal reduces these differences (Akiyoshi et al., 2018).

To further test microglial‐dependent differences in basal neuronal transmission across the light/dark cycle, we measured the paired pulse ratio (PPR) at 50 ms intervals, a form of short‐term plasticity related to neurotransmitter release probability, by extracellular field recordings in hippocampal CA1 region. We observed a main effect of treatment (*F*[1, 79] = 11.159, *p* = .001) whereas that of phase was not significant (*p* = 0.315). In particular, post hoc analysis revealed that, at ZT16, microglia depletion increased the PPR value compared to control (ZT16: PPR = 1.07 ± 0.02, *n*/*N* = 31/16; ZT16 PLX5622: PPR = 1.18 ± 0.04, *n*/*N* = 17/7, *t* = 3.012, *p* = .004, Figure 2d), suggesting a reduced probability of glutamate release in the dark phase. In the light phase, PPR value was not modified by PLX5622 treatment (ZT4: PPR = 1.12 ± 0.03, *n*/*N* = 17/9; ZT4 PLX5622: PPR = 1.19 ± 0.03, *n*/*N* = 16/7, *t* = 1.81, *p* = 0.074; Figure 2d). These results further indicate that microglia, in the dark phase, contribute to the modulation of glutamate release.

We also explored the post‐synaptic plasticity processes in the CA1 hippocampal region by recording LTP evoked by two spaced (30 min apart) Schaffer collateral high‐frequency stimulation (HFS) and analyzing the field excitatory postsynaptic potential (fEPSP) 20 min after each stimulation. In particular, we recorded LTP during the dark phase, at ZT16, and the light phase, at ZT4, in control and PLX5622 treated mice. Following the first stimulation, the main effect of treatment and phase emerged (*F*[1, 96] = 5.407, *p* = .022; *F*(1, 96) = 6.791, *p* = .011, respectively). Similarly, after the second stimulation, we found a significant main effect of phase (*F*[1, 82] = 9.433, *p* = .003), whereas those of treatment were not significant (*F*[1, 82] = 3.908, *p* = .052). Post hoc analysis revealed that after the first train, in control conditions, LTP amplitude did not show significant differences between ZT4 and ZT16 (ZT4: 1.32 ± 0.02 mV, *n*/*N* = 26/12; ZT16: 1.29 ± 0.02 mV, *n*/*N* = 46/23 *p* = .316, Figure S6A, B), indicating that hippocampal LTP is not affected by the light/dark cycle. By contrast, in microglia‐depleted mice, LTP amplitude was higher at ZT4 compared to ZT16 (ZT4: 1.46 ± 0.06 mV, *n*/*N* = 12/7; ZT16: 1.34 ± 0.04 mV, *n*/*N* = 14/8; *p* = .016, Figure S6A, B), being at ZT16 similar and at ZT4 enhanced compared to control (*p* = .551 and *p* = .012, respectively). Similarly, after the second stimulation, LTP amplitude in control group did not show significant difference between the two phases (ZT4:1.50 ± 0.04 mV, *n*/*N* = 22/11; ZT16: 1.43 ± 0.04 mV, *n*/*N* = 38/21, *p* = .229, Figure S6A,B), whereas microglial depletion caused an increase of LTP amplitude at ZT4 compared to ZT16 (ZT4: 1.68 ± 0.09 mV, *n*/*N* = 11/7; ZT16: 1.42 ± 0.064 mV, *n*/*N* = 13/8, *p* = .005, Figure S6A,B). Moreover, at ZT4, the LTP amplitude of PLX5622 treated mice resulted increased respect to control (*p* = .016).

These results suggest that in basal condition LTP amplitude do not differ between ZT4 and ZT16. By contrast, microglia depletion uncovers an inhibitory role of microglia during the light phase, being the LTP level increased by PLX5622 treatment.

### 3.3 Microglial *cx3cr1* expression decreases during the light period

**FIGURE 3:**
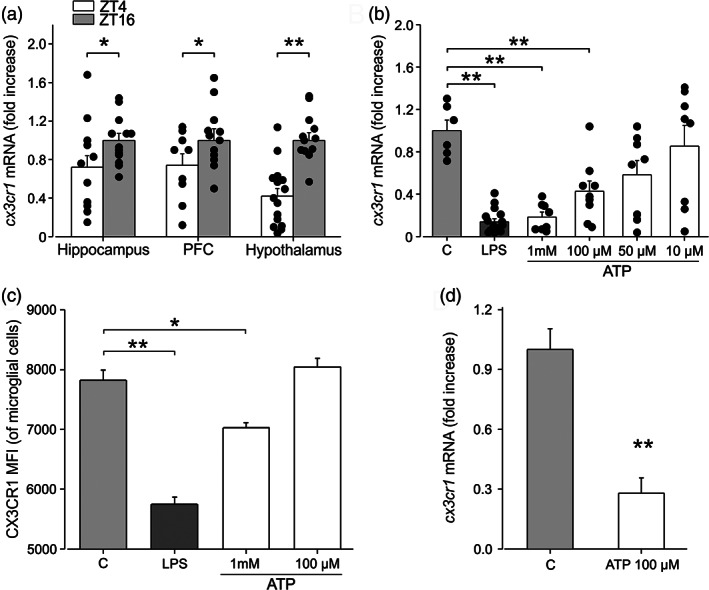
Microglial expression of *cx3cr1* decreases during the light phase and upon ATP stimulation in vitro. (a) *cx3cr1* mRNA expression analysis by RT‐PCR in CD11b + cells obtained at ZT4 and ZT16 from hippocampus (*n* = 12 and 13 mice respectively), PFC (*n* = 9 and 12 mice respectively) and hypothalamus (*n* = 15 and 13 mice respectively) and individual distribution of data. Data are expressed as *cx3cr1* mRNA fold increase normalized to *gapdh* expression; values shown are normalized to ZT16. cx3cr1 mRNA levels are increased during dark in all regions analyzed. Data are expressed as mean ± SEM. * *p* = .034 (hippocampus) and *p* = .03 (PFC); ** *p* < .001 (one‐way ANOVA). (b) *cx3cr1* mRNA expression analysis by RT‐PCR in primary wild type microglia obtained from pups treated for 4 h with LPS (100 ng/ml) and ATP at different concentrations. LPS treatment and ATP at 1 and 100 μM decrease cx3cr1 mRNA levels. Data are expressed as *cx3cr1* mRNA fold increase normalized to *gapdh* expression; values shown are normalized to vehicle (c). Data are expressed as the mean ± SEM. ** *p* < .001 (one‐way ANOVA, followed by Dunn's post hoc test). (c) Median fluorescence intensity (MFI) of CX3CR1 staining is decreased in primary wild type microglia stimulated for 4 h with LPS (100 ng/ml) and ATP 1 mM and 100 μM. Data are expressed as mean ± SEM of the MFI. ** *p* < .001 C versus LPS, * *p* = 0.006 C versus ATP 1 mM, one‐way ANOVA followed by Holm‐Sidak post hoc test. (d) *cx3cr1* mRNA expression by RT‐PCR is also decreased in primary wild type microglia obtained from adult mice following 4 h of ATP (100 μM) treatment. Data are expressed as mean ± SEM of *cx3cr1* mRNA fold increase normalized to *gapdh* expression; values shown are normalized to vehicle (c). ** *p* < 0.001 (Student's *t*‐test)

Pieces of evidence describe the role of the CX3CL1/CX3CR1 pair in modulating neuron to microglia communication (reviewed in Limatola and Ransohoff, 2014; Trettel et al., 2020), as well as in regulating synapse morphology and functioning (Zhan et al., 2014; Paolicelli et al., 2014). We investigated whether the diurnal cycle affected microglial expression of CX3CR1, which is involved in communication with neuronal cells. With this aim, we performed RT‐qPCR analysis on enriched microglial population (CD11b+ cells) isolated from the brain of mice at ZT4 and ZT16, to analyze *cx3cr1* expression. Data shown in Figure 3a indicate that three brain regions analyzed, *cx3cr1* expression was significantly reduced during the light period (hippocampus: ZT16: 1 ± 0.07, *N* = 13; ZT4: 0.72 ± 0.12, *N* = 12, *p* = .034; PFC: ZT16: 1 ± 0.12 *N* = 12; ZT4 0.74 ± 0.11, *N* = 9, *p* = .03; hypothalamus: ZT16: 1 ± 0.08, *N* = 13; ZT4: 0.42 ± 0.08, *N* = 15, *p* < .001 One‐way ANOVA), suggesting a phase‐dependent modulation of neurons‐microglia communication through this signaling pathway.

Since it has been reported that brain levels of ATP increase during the sleep phase (Dworak et al., 2010), we stimulated primary microglial cultures with different concentrations of ATP to investigate the mechanisms involved in the light‐dependent modulation of *cx3cr1* expression. Results presented in Figure 3b show that extracellular ATP reduces *cx3cr1* expression in primary microglia obtained from pups in a concentration‐dependent way. LPS was used as positive control for *cx3cr1* modulation (as reported by Boddeke et al., 1999; Wynne et al., 2010) (C: 1 ± 0.10, *n*/*N* = 6/3 vs. LPS: 100 ng/ml 0.14 ± 0.03, *n*/*N* = 16/3, *p* < .001; ATP 1 mM: 0.19 ± 0.05, *n*/*N* = 8/3, *p* < .001; ATP 100 μM: 0.43 ± 0.10, *n*/*N* = 9/3, *p* = .01; ATP 50 μM: 0.58 ± 0.14, *n*/*N* = 8/3, *p* = .17; ATP 10 μM: 0.85 ± 0.19; *n*/*N* = 8/3, *p* = .99; one‐way ANOVA followed by Dunn's post hoc test). We also investigated CX3CR1 receptor expression upon extracellular ATP stimulation in cultured microglial cells by FACS analysis. Figure 3c shows that ATP reduces the expression of CX3CR1, confirming the reduction of *cx3cr1* mRNA expression (C: 7822 ± 171 vs. ATP 1 mM: 7029 ± 83, *p* = .006; ATP 100 μM: 8043 ± 144; *n*/*N* = 3/3 per condition; *p* = .27, one‐way ANOVA followed by Holm‐Sidak post hoc test). LPS was again used as positive control (LPS 100 ng/ml: 5745 ± 122, *n* = 3/3; C vs. LPS, *p* < .001). Moreover, we confirmed the reduction of *cx3cr1* expression due to ATP stimulation on microglial cells obtained from adult mice, as shown in Figure 3d (C: 1 ± 0.1; ATP 100 μM: 0.28 ± 0.07; *n*/*N* = 3/3; *p* < .001, Student's *t*‐test). These results led us to speculate that the increased levels of ATP present in the brain in the first hours of sleep, due to reduced use of energy during the NREM phase (Dworak et al., 2010), could reduce *cx3cr1* expression in microglia, thus affecting their response to neuronal CX3CL1 signaling.

### 3.4 CX3CR1‐CX3CL1 signaling underlies microglia effects along the light/dark cycle

To investigate the role played by CX3CR1 signaling in mediating microglial effects on the light/dark cycle, we extended our study to the *cx3cr1* ^*GFP/GFP*^ mice. For these experiments, C57BL/6J mice were used as control, due to the similarity in the genetic background.

**FIGURE 4:**
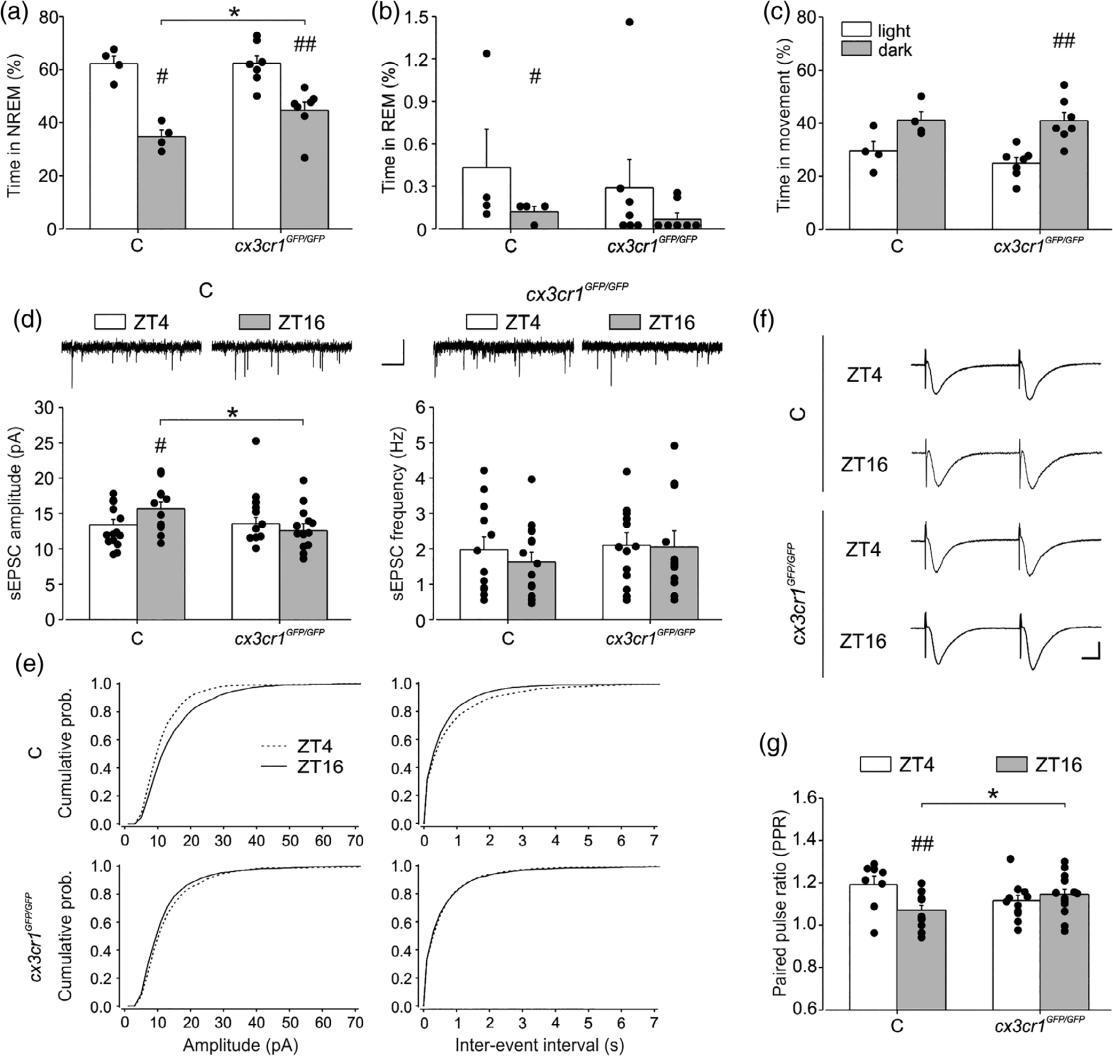
*c3cr1* deficiency effects on sEPSC and video‐EEG recordings. Mean ± SEM and individual distribution of non‐rapid eye movement (NREM) sleep (a), non‐rapid eye movement (REM) (b) sleep and movement (c) duration in 7 h of light versus 7 h of dark in cx3cr1GFP/GFP mice (*n* = 7) and control mice (C; *n* = 4). The within‐group statistical analysis was performed by Wilcoxon test for paired measures and showed statistical differences for NREM sleep (C # *p* = .03 uncorrected, *z* = 1.83, *t* = 0.00; cx3cr1GFP/GFP ## *p* = .0089 uncorrected, *z* = 2.36, *t* = .00), REM sleep (C # *p* = .04 uncorrected, *z* = 1.10, *t* = 2.00) and movement duration (cx3cr1GFP/GFP ## *p* = 0.0089 uncorrected, *z* = 2.36, *t* = 0.00), in light versus dark conditions. The between‐group statistical analysis was performed by Mann–Whitney test for unpaired measures and showed increased NREM sleep (* *p* = 0.04 uncorrected, *z* = 1.80, *U* = 4.00) in *cx3cr1* ^*GFP/GFP*^ mice respect to (c,d) representative traces of spontaneous EPSC (top) and histograms for the mean amplitude and frequency (bottom) in controls (C) and *cx3cr1* ^*GFP/GFP*^ mice at ZT4 and ZT16. The amplitude of sEPSC is increased in C during dark (ZT16 C: 12/3 cells/mice, ZT4 C: 15/3, *t* = 2.084, *p* = 0.043) and reduced in *cx3cr1* ^*GFP/GFP*^ mice in dark (13/3, *t* = 2.084, *p* = .042). Scale bars: 1 s (horizontal), 20 pA (vertical). (e) Cumulative probability function for sEPSCs amplitudes compared at the two daily times in controls (ZT4:1121 and ZT16:1267 events) showed a rightward shift at ZT16 (KS, *p* < .0001, top left). The IEI cumulative function is shifted to the left in C at ZT16 indicating an increased sEPSC frequency (KS, *p* = .0036, top right). No shift is detected in *cx3cr1* ^*GFP/GFP*^ mice for sEPSC amplitude and IEI at the two time points considered (KS, *p* = .748 and *p* = .503, bottom). (f) Representative fEPSP traces for PPR experiments performed (ISI = 50 ms) at ZT4 and ZT16 in controls and *cx3cr1* ^*GFP/GFP*^. (g) Histogram of the mean values of PPR at ZT4 and ZT16 in control (C, ZT4: 8/4 and ZT16: 13/5 slices/mice) and *cx3cr1* ^*GFP/GFP*^ mice (ZT4: 8/4 and ZT16: 15/6 slices/mice). Paired‐pulse ratio values are decreased in control (*t* = 2.924, *p* = .005) and increased in *cx3cr1* ^*GFP/GFP*^ mice (*t* = 2.144, *p* = .038) at ZT16. Scale bars: 0.3 mV (vertical), 10 ms (horizontal). Data are shown as mean ± SEM statistical analysis was performed with two‐way ANOVAs, holm‐Sidak post hoc comparison. *, # *p* < .05, **, ## *p* < .01. Cumulative probability functions were compared with Kolmogorov–Smirnov test

We first investigated sleep duration in the light/dark cycle. As for the PLX5622 treated mice, the *cx3cr1* ^*GFP/GFP*^ mice showed longer NREM sleep durations during the dark phase (44.6 ± 3.2%) in comparison with the control group (34.7% ± 2.49%) (Figure 4a *p* = .04 uncorrected, and Table 1 and Tables S1–S3). Similar results were obtained also considering the total sleep (NREM + REM; *p* = .035 uncorrected). For the REM sleep, control mice showed significant difference between the light and dark (*p* = .04 uncorrected), whereas no differences in REM sleep were observed for *cx3cr1* ^*GFP/GFP*^ (*p* = .27) (Figure 4b). No significant differences were observed in the amount of time in movement during the dark phase in *cx3cr1* ^*GFP/GFP*^ mice (40.91% ± 3.12%) in comparison with the control group (41.08% ± 3.17%) (Figure 4c *p* = .5).

Overall, these data confirm a relevant role of microglia in sleep duration along the daily cycle, highlighting the involvement of CX3CR1 signaling pathway. We then recorded synaptic transmission in mice lacking *cx3cr1*. When analyzing sEPSC amplitude, we did not find a main effect of genotype and phase, but a statistically significant interaction between the two factors emerged (*p* = .021). In particular, Holm‐Sidak post hoc analysis revealed that in control mice the mean sEPSC amplitude was higher in the dark (ZT16: 15.68 ± 0.96 pA *n*/*N* = 12/3, ZT4: 13.21 ± 0.86 pA, *n*/*N* = 15/3, *t* = 2.084, *p* = .043, Figure 4d, left) with a rightward shift of the cumulative distribution compared to light condition (KS test *p* < .0001, Figure 4e, top left). Interestingly, in *cx3cr1* ^*GFP/GFP*^ mice sEPSC amplitude was similar at ZT4 and ZT16 (ZT4: 14.86 ± 0.96 pA, *n*/*N* = 12/3; ZT16: 12.901 ± 0.92 pA, *n*/*N* = 13/3 cells, *t* = 1.468, *p* = 0.149 Figure 4d, right) with only a minor leftward shift of the cumulative distribution function during light condition (KS *p* = 0.003 Figure 4e, bottom left), recapitulating what observed in PLX5622‐treated mice. In addition, at ZT16 *cx3cr1* ^*GFP/GFP*^ mice showed a significant reduction of sEPSC amplitude compared to control mice (*t* = 2.084, *p* = .042) as observed in microglia depleted mice (see Figure 2) Figure 4d, left). In C57BL/6J, we did not observe a difference in the mean sEPSC frequency at light (ZT4:1.76 ± 0.32 Hz) compared to the dark (ZT16: 2.08 ± 0.34 Hz, *t* = 0.675, *p* = .503, Figure 4d, right); however, the cumulative distribution curve for IEI was significantly shifted toward the left at ZT16 (KS *p* = .0036, Figure 4e, top right). In *cx3cr1* ^*GFP*/GFP^ mice, we found no difference either for the mean values at ZT4 (2.05 ± 0.36 Hz) and ZT16 (2.05 ± 0.34 Hz, *t* = 0.000727, *p* = .99, Figure 4d, right) and the cumulative functions for IEI (KS *p* = .748) (Figure 4e, bottom right).

When we measured short‐term plasticity, a significant interaction between genotypes and phases emerged (*p* = .008) and post hoc analysis revealed that in the dark PPR was higher in *cx3cr1* ^*GFP/GFP*^ compared to control (ZT16 *cx3cr1* ^*GFP/GFP*^: 1.14 ± 0.02, *n*/*N* = 15/6; ZT16 CTRL: 1.07 ± 0.03, *n*/*N* = 13/5, *t* = 2.144, *p* = .038, Figure 4f,g), whereas in the light phase it was similar, as observed in microglia‐depleted mice (ZT4 *cx3cr1* ^*GFP/GFP*^: 1.11 ± 0.02, *n*/*N* = 12/5; ZT4 CTRL: 1.19 ± 0.04, *n*/*N* = 8/4, *t* = 1.803, *p* = .078) (see Figure. 2d). Of note, at difference with C57BL/6N, in C57BL/6J control mice the PPR at ZT4 was increased respect to ZT16 (ZT16: 1.07 ± 0.03, *n*/*N* = 13/5; ZT4: 1.19 ± 0.03, *n*/*N* = 8/4, *t* = 2.924, *p* = .005), indicating PPR differences in the two mouse strains.

In addition, we investigated long‐term plasticity also in *cx3cr1* ^*GFP/GFP*^. LTP amplitude of the transgenic mice at ZT4 and ZT16 did not show significant differences (ZT4: 1.41 ± 0.03 mV, *n*/*N* = 25/10; ZT16: 1.39 ± 0.04 mV, *n*/*N* = 22/10; *p* = 0.558, Figure S6C, D). However, at ZT4, LTP amplitude of *cx3cr1* ^*GFP/GFP*^ mice was significantly increased compared to control (*p* = .037) similarly to what observed in PLX5622 treated mice (see Figure S6A, B). Comparably, after the second stimulation, LTP amplitude analyzed in control and *cx3cr1* ^*GFP/GFP*^ mice did not show significant difference between the two phases (for control: ZT4: 1.41 ± 0.07 mV, *n*/*N* = 9/6; ZT16: 1.51 ± 0.06 mV, *n*/*N* = 15/9; *p* = .333; for *cx3cr1* ^*GFP/GFP*^: ZT4: 1.61 ± 0.05 mV, *n*/*N* = 19/9; ZT16: 1.53 ± 0.05 mV, *n*/*N* = 18/8; *p* = 0.253, Figure S6C, D). However, at ZT4, LTP amplitude of *cx3cr1* ^*GFP/GFP*^ mice was significantly increased compared to control (*p* = .03), resembling what was observed in microglial‐depleted mice.

Altogether, these data indicate that *cx3cr1* participates in regulating the phase‐dependent microglial alterations.

## 4 DISCUSSION

In this study, we investigated the role of microglia in the changes associated to sleep/wake phases throughout the 12:12 light/dark cycle, focusing on the modulation of hippocampal synaptic functions and on behavioral states upon microglial depletion with CSF‐1R inhibitor. We demonstrate for the first time that the microglial depletion with PLX5622 increases the time spent by mice in the NREM sleep during the dark phase. Upon microglia depletion, we also reported a reduction in the hippocampal excitatory neurotransmission in the dark and an increased long‐term potentiation during the light phase indicating that microglia cells play a role in shaping neuronal functions along the daily cycle.

In line with previous data reporting post‐synaptic changes occurring during wakefulness, such as increased levels of hippocampal AMPA receptor subunits and ultra structural spine modifications (Vyazovskiy et al., 2008 Liu et al., 2010; Yang et al., 2014; de Vivo et al., 2017), we found an increased amplitude of both miniature and sEPSC events recorded in the CA1 hippocampal region of mice at ZT16. In addition, at ZT16, we measured an increase in sEPSC frequency and a parallel reduction in the PPR, indicating that also presynaptic changes occur along the daily cycle that can be ascribed to modification of the probability of glutamate release (Zucker, 1989). Overall, our data support the idea of a net synaptic potentiation taking place during wakefulness in the hippocampus, in accordance with the synaptic homeostasis hypothesis (SHY) that proposes sleep as a period necessary to maintain a proper control of synaptic strength in the brain (Tononi and Cirelli, 2003).

Our data that microglial depletion with PLX5622 reduced the amplitude of both mEPSC and sEPSC at ZT16, abolishing the phase‐dependent differences in basal synaptic transmission, suggest that microglial cells predominantly control the synaptic strength during the wake phase. Previous studies demonstrated that the direct contact between microglial processes and synapses in awake mice reflects enhanced synaptic activity and that partial microglial ablation decreased the synchronization of evoked neural activity (Akiyoshi et al., 2018). In accordance with our findings, microglial depletion in rats has been associated with decreased total hippocampal synaptic transmission and impaired cognitive function (Yegla et al., 2021). On the other hand, it has been also reported that chronic microglial depletion increases both excitatory and inhibitory synaptic connections to excitatory cortical neurons (Liu et al., 2021). This evidence, together, concurs to highlight a prominent role of microglia in sculpting neuronal circuit connectivity and regulating subsequent functional activity in a region‐specific manner.

Microglial depletion with PLX5622 is an efficient and reversible event (Elmore et al., 2014; Dagher et al., 2015) but recent evidence showing that, in addition to depleting microglia, PLX5622 also induces long‐term changes in the peripheral myeloid and lymphoid cells must be considered (Lei et al., 2020). Furthermore, it has been shown that not the entire population of microglia responds to CSF‐1R antagonists and a small percentage persists with depletion, which could be responsible for some of the observed effects (Elmore et al., 2014).The fractalkine receptor CX3CR1 has a key role in mediating the communication among neurons and microglial cells, in physiological and pathological conditions (Maggi et al., 2011; Limatola and Ransohoff, 2014). We report that, similarly to what observed upon microglial depletion, *cx3cr1* ^*GFP/GFP*^ mice had no phase‐dependent differences in sEPSC amplitude, indicating that the fractalkine/CX3CR1 signaling between neurons and microglia is necessary for the wake‐dependent increase of excitatory synaptic transmission. Similarly, we observed a reduction in the frequency of sEPSC and a corresponding increase of the PPR at ZT16 both in microglial‐depleted and in *cx3cr1* ^GFP/GFP^ mice, suggesting that, during wake, microglia positively modulate presynaptic glutamate release probably trough the CX3CR1 signaling.

Several evidence demonstrated that sleep is relevant for initial memory encoding and subsequent long‐term memory consolidation, exerting effects on molecular, cellular and network mechanisms of plasticity (Abel et al., 2013, Klinzing et al. 2019). Hippocampal synaptic plasticity may vary with the time of day: diurnal variations in the incidence and magnitude of LTP in the CA1 region have been reported, with the largest responses occurring in the day (Harris and Teyler, 1983; Raghavan et al., 1999) or in the night (Chaudhury et al. 2005, Jilg et al., 2019; Dana and Martinez Jr, 1984; Leung et al., 2003). The variability of these results can be related to the different experimental conditions, such as stimulation protocol and animal model used, but also to the strong dependence of plasticity to the phase of sleep (NREM vs. REM) which are difficult to assess in ex vivo preparations. For these reasons, our results on the alterations of hippocampal LTP upon microglial alterations in the dark and light phase must be interpreted considering these experimental limitations. In control conditions, we report no differences in LTP magnitude at ZT4 and ZT16. However, both microglial depletion and CX3CR1 deletion, induce an increase of LTP amplitude at ZT4, as previously reported (Maggi et al., 2011). It can be speculated that interfering with microglia during this period affects the production of cytokines such as interleukin‐1 and tumor necrosis factor‐α which have been associated to NREM induction (Hide et al., 2000; Shieh et al., 2014) and LTP inhibition (Prieto et al., 2019). Although further experiments are necessary to finely define microglial function on long term synaptic plasticity along the circadian cycle, our data demonstrate that microglia differentially affect LTP and synaptic strength in the light/dark phases.

Previous data demonstrated that hampering microglial activities with minocycline in aged rats (Griffin et al., 2006) or impairing fractalkine signaling in the *cx3cr1* knockout mice (Maggi et al., 2011; Milior et al., 2016) potentiated the hippocampal synaptic plasticity. Together, these data indicate that the phase‐dependent microglial effects on hippocampal neurotransmission are mediated via the expression of *cx3cr1*.

Microglial depletion with PLX5622 or *cx3cr1* deletion results in alteration of NREM phase duration and reduced excitatory neurotransmission in the dark, likely reflecting a key role for *cx3cr1* expressing microglial cells for the homeostasis of these functions. Different results on sleep duration have been previously reported when microglial activity is acutely perturbed with the drug minocycline, also in conditions of sleep deprivation (Wisor et al., 2011). These mixed findings may be explained by alternative off‐target effects of minocycline, or by distinct functional states of microglia in the different experimental conditions.

We observe that the depletion of microglia did not affect REM sleep. Even if we cannot provide experimental explanations for this specific effect of microglial cell depletion, we could speculate that microglial cells mostly impact on circuits involved in wake during the active phase, such as the hypothalamic orexin and MHC neurons (Lee et al., 2005; Hassani et al., 2009). Further experiments are needed to better understand these differences.

We hypothesize that the reduction of *cx3cr1* expression in microglial cells isolated from three different brain regions of mice at ZT4 could be explained by a direct effect of ATP accumulated in the brain during sleep (Dworak et al., 2010; Krueger et al., 2010; Marpegan et al., 2011). This hypothesis is supported by data showing that, in vitro, ATP administration decreases *cx3cr1* expression in microglia. The accumulation of ATP during sleep has been associated with the need to replenish the brain energy sources (Burkeen et al., 2011). ATP is also released during neuronal activity and it is a recognized danger signal for brain parenchyma (Rodrigues et al., 2015). Extracellular ATP can reach high local concentrations, especially at regions of cell‐to‐cell contact (Pankratov et al., 2006), but it must be considered that ATP is rapidly degraded with the production of adenosine, which is a very well described sleep regulator (Porkka‐Heiskanen et al., 1997; Stenberg et al., 2003). A recent report describes an ATP‐dependent regulation of adenosine‐mediated microglial depression of neuronal firing (Badimon et al., 2020). We hypothesize that the ATP‐induced reduction of *cx3cr1* expression during the light phase represents the activation of coordinated communication mechanisms among astrocytes‐microglia and neuronal cells to re‐tune synaptic activities and the strength of circuitry in the different day phases to allow for learning, memory and environmental adaptation.

We show that PLX5622 treatment had minor effects on motor activity and only during the light phase. Previous studies reported that microglial depletion did not alter behavior, locomotor, or cognitive activity in mice, but improved cognition in the 3xTg‐AD mouse model of Alzheimer's disease and in model of cranial irradiation (Elmore et al., 2014; Dagher et al., 2015; Acharya et al., 2016). Other studies report that altered cerebellar CSF‐1R signaling affects motor learning and social interaction (Kana et al., 2019). The different outcomes on motor functions could be explained by the different experimental conditions and by the long‐ versus short‐term analysis performed to detect alterations of basal motor activities.

In this study, we did not observe specific effects of microglial depletion on circadian internal rhythms, as demonstrated by the absence of alteration of specific behavioral parameters such as the mean activity, the activity onset, and the phase shift in a dark/dark housing condition. This led us to speculate that the effects of microglial depletion on total sleep and NREM sleep duration are due to modulation of homeostatic processes involved in the control of the sleep/wake phase rather that circadian mechanisms.

To conclude, several studies investigating the role of microglial cells in the sleep/wake cycle focused on the effects induced by conditions of sleep deprivation, with different experimental approaches, from gentle handling to forced stimulation of laboratory animals (Bellesi et al., 2017; Wadhwa et al., 2017; Tuan and Lee 2019; Hall et al., 2020). Keeping animals awake involves the activation of the hypothalamic–pituitary–adrenal axis, thus contributing to different levels of stress; this must be considered as additional variable together with sleep loss to decipher the observed effects (Meerlo et al., 2002; Havekes and Aton, 2020). In this study, we demonstrate that microglia participate in the regulation of sleep/wake cycle and synaptic transmission in a phase‐dependent manner. Previous studies reported microglial alteration upon chronic sleep deprivation protocols (Bellesi et al., 2017), hypothesizing that prolonged sleep loss, priming microglial cells, could increase the brain susceptibility induced by a secondary insult. The variations in *cx3cr1* expression across different zeitgeber times suggest that the functional interactions of microglia with hippocampal synapses vary along the light/dark cycle. The observation that the lack of *cx3cr1* parallels the depletion of microglia on sleep and synaptic transmission strongly indicates that the CX3CR1/CX3CL1 signaling pathway is crucial for the homeostatic role of microglia in the sleep/wake cycle. Our data led us to speculate that microglial cells, through CX3CR1 signaling, participate in reshaping the glutamatergic synaptic transmission with pre‐ and postsynaptic mechanisms, contributing to the synaptic up scaling taking place during wake. Further investigations will be necessary to understand how CX3CR1 mediates these effects.

Additional studies targeting the sleep pressure and the circadian processes will be also necessary to clarify the detailed neurophysiological mechanisms underlying these effects.

## CONFLICT OF INTEREST

The authors declare no conflict of interest.

## Supporting information


**Appendix S1.** Supporting Information.

**Figure S1** Effectiveness of PLX5622 treatment in depleting microglia. Representative images showing IBA1‐stained cells in PFC, hypothalamus and hippocampus for controls (upper panels) and mice treated with PLX5622 for at least 7 days (lower panels). Scale bars: PFC 200 mm; hypothalamus 300 mm; and hippocampus 350 mm.

**Figure S2** Effect of microglial depletion on NREM sleep duration in the dark and light phases. A. Mean ± s.e.m. and individual distribution of NREM sleep duration in 22 h of light vs 22 h of dark in C57BL/6N (C) and PLX5622‐treated mice. (C light: 50.27 ± 1.57%, C dark: 33.45 ± 3.79%, n = 5, p = 0.02 corrected, z = 2.00, t = 0.00; PLX5622 light: 52.73 ± 2.37%, PLX5622 dark: 44.96 ± 1.41%, n = 5, p = 0.02 corrected, z = 2.00, t = 0.00, Wilcoxon test; light: C vs PLX5622, p = 0.26, z = 0.6, U = 9.0; dark: C vs PLX5622, p = 0.018 corrected, z = −2.1, U = 2.0, Mann–Whitney test). **B**. Time‐courses of NREM sleep. Data are expressed as a percentage of the total time analyzed and are shown as mean ± s.e.m.

**Figure S3** Motor activity and circadian variables recorded with DVC in light–dark condition. A. Color‐coded heatmaps for control (C, top) and PLX5622 group (bottom) showing the raw activity recorded by DVC for 5 days under light–dark condition. B. Average of hourly activity over 5 days in control (C, black squares, n = 18) and PLX5622 (white squares, n = 15); s.e.m. are omitted for graphical clarity. C. Hourly activity averaged over 5 days for control (C,) and PLX5622‐treated mice. D. Five days time course analysis of activity along 12 h of light (top) and 12 h of dark (bottom). No significant difference was observed between the two groups in light and dark condition (p > 0.05, Two‐way ANOVA for repeated measures). E. Mean of the acrophase (C: 16.16 ± 0.39 CT; PLX5622 16.70 ± 0.37 CT, p = 0.33, Student's t‐test). F. diurnality for control (C) and PLX5622 groups (C: 26.10 ± 2.21%; PLX5622 22.04 ± 1.30%, p = 0.14, Student's t‐test). Data are shown as mean ± s.e.m.

**Figure S4** Motor activity and circadian variables recorded with DVC in dark–dark condition. A. Color‐coded heatmaps for control (C, top) and PLX5622 group (bottom) showing the raw activity recorded by DVC for 5 days of total darkness. B. Average of hourly activity over 5 days in control (C, black squares, n = 6) and PLX5622 (white squares, n = 5); s.e.m. are omitted for graphical clarity. C. Phase shift for control (C) and PLX5622‐treated mice in constant darkness (C: −15.46 ± 1.51 CT; PLX5622–14.99 ± 2.10 CT p = 0.85, Student's t‐test, days 8–26). D. Time course analysis of activity in constant darkness along CT 7–19 (top) and CT 19–7 (bottom). No significant difference was observed between the two groups in light and dark condition (p > 0.05, Two‐way ANOVA for repeated measures) E. Mean of the acrophase (C: 13.50 ± 1.10 CT; PLX5622 15.06 ± 1.77 CT, p = 0.46, Student's t‐test) and F. diurnality for control (C) and PLX5622 groups (C: 46.50 ± 2.19%; PLX5622 44.28 ± 4.44%, p = 0.64, Student's t‐test). Data are shown as mean ± s.e.m.

**Figure S5** Effect of microglial depletion with PLX3397 on sleep and wake duration in the dark and light phases. A‐C. Time spent in NREM (A), REM (B) and Wake (C) during the light and the dark period by the mice before (C) and after microglial depletion (PLX3397). NREM: light C 63.23 ± 0.96, dark C 28.51 ± 1.5%, light PLX3397 64.49 ± 1.11%, dark PLX3397 38.89 ± 4.34%. REM: light C 6.61 ± 0.29, dark C 2.12 ± 0.2%, light PLX3397 5.85 ± 0.30%, dark PLX3397 2.43 ± 0.17%; WAKE: light C 27.36 ± 0.94, dark C 68.23 ± 1.63%, light PLX3397 26.75 ± 1.19%, dark PLX3397 57.04 ± 4.66%. D‐F. Hourly percentage of time spent in NREM (D), REM (E) and wake (F) over 24 hours by the mice before (C) and after microglial depletion (PLX3397). Data are expressed as percentage of the total time analyzed and are shown as mean ± s.e.m. ## p < 0.01 (Wilcoxon test, p < 0.005 = 0.05 corrected); ** p < 0.01, * p = 0.017 (Mann‐Withney test, p < 0.005 = 0.05 corrected). ## p < 0.001 (Two‐way ANOVA, repeated measures, followed by Bonferroni post‐hoc test).

**Figure S6** Hippocampal CA1 LTP is affected by PLX5622 and *cx3cr1* deletion only in the light phase. A. Representative fEPSP traces for LTP recorded in control (C) and PLX5622 conditions at ZT4 and ZT16 during baseline, at 30 min (post‐LTP 1) and at 60 min (post‐LTP 2); scale bars: 0.3 mV (vertical), 5 ms (horizontal). B. In control conditions (C), LTP amplitude was similar at ZT4 and ZT16 (white and gray squares, respectively) following both the first and second tetanus. PLX5622 treatment causes an increase of LTP amplitude at ZT4 compared to ZT16 (white and gray circles, respectively; * p = 0.016, ** p = 0.005, PLX5622 ZT4 vs ZT16). Arrows indicate time of application of HFS (100 Hz trains of 1 sec duration, 30 min apart). C. Representative fEPSP traces for LTP recorded in control (C) and *cx3cr1* ^*GFP/GFP*^ mice at ZT4 and ZT16 during baseline, at 30 min (post‐LTP 1) and at 60 min (post‐LTP 2); scale bars: 0.3 mV (vertical), 5 ms (horizontal). D. In control conditions (C), LTP amplitude was similar at ZT4 and ZT16 (white and gray squares, respectively) following both the first and second tetanus. *cx3cr1* deletion causes an increase of LTP amplitude at ZT4 compared to ZT16 (white and gray circles, respectively; first train: * p = 0.037, second train: * p = 0.03, *cx3cr1* ^*GFP/GFP*^ ZT4 vs ZT16). Arrows indicate time of application of HFS (100 Hz trains of 1 sec duration, 30 min apart). Data are shown as mean ± s.e.m.

## Data Availability

All data are available in the main text or the supplementary materials. The data that support the findings of this study are available from the corresponding author upon reasonable request.

## References

[glia24090-bib-0001] Abel, T. , Havekes, R. , Saletin, J. M. , & Walker, M. P. (2013). Sleep, plasticity and memory from molecules to whole‐brain networks. Current Biology, 23(17), 774–788. 10.1016/j.cub.2013.07.025 PMC4263505 24028961

[glia24090-bib-0002] Acharya, M. M. , Green, K. N. , Allen, B. D. , Najafi, A. R. , Syage, A. , Minasyan, H. , Le, M. T. , Kawashita, T. , Giedzinski, E. , Parihar, V. K. , West, B. L. , Baulch, J. E. , & Limoli, C. L. (2016). Elimination of microglia improves cognitive function following cranial irradiation. Scientific Reports, 6, 31545. 10.1038/srep31545 27516055 PMC4981848

[glia24090-bib-0094] Akiyoshi, R. , Wake, H. , Kato, D. , Horiuchi, H. , Ono, R. , Ikegami, A. , Haruwaka, K. , Omori, T. , Tachibana, Y. , Moorhouse, A. J. , & Nabekura, J. (2018). Microglia enhance synapse activity to promote local network synchronization. eNeuro, 5(5), ENEURO.0088–18.2018. 10.1523/eneuro.0088-18.2018 PMC6220592 30406198

[glia24090-bib-0003] Badimon, A. , Strasburger, H. J. , Ayata, P. , Chen, X. , Nair, A. , Ikegami, A. , Hwang, P. , Chan, A. T. , Graves, S. M. , Uweru, J. O. , Ledderose, C. , Kutlu, M. G. , Wheeler, M. A. , Kahan, A. , Ishikawa, M. , Wang, Y. C. , Loh, Y. E. , Jiang, J. X. , Surmeier, D. J. , … Schaefer, A. (2020). Negative feedback control of neuronal activity by microglia. Nature, 586(7829), 417–423. 10.1038/s41586-020-2777-8 32999463 PMC7577179

[glia24090-bib-0005] Bellesi, M. , de Vivo, L. , Chini, M. , Gilli, F. , Tononi, G. , & Cirelli, C. (2017). Sleep loss promotes astrocytic phagocytosis and microglial activation in mouse cerebral cortex. The Journal of Neuroscience, 37(21), 5263–5273. 10.1523/JNEUROSCI.3981-16.2017 28539349 PMC5456108

[glia24090-bib-0007] Boddeke, E. W. , Meigel, I. , Frentzel, S. , Biber, K. , Renn, L. Q. , & Gebicke‐Härter, P. (1999). Functional expression of the fractalkine (CX3C) receptor and its regulation by lipopolysaccharide in rat microglia. European Journal of Pharmacology, 374(2), 309–313. 10.1016/s0014-2999(99)00307-6 10422773

[glia24090-bib-0008] Bolós, M. , Perea, J. R. , Terreros‐Roncal, J. , Pallas‐Bazarra, N. , Jurado‐Arjona, J. , Ávila, J. , & Llorens‐Martín, M. (2017). Absence of microglial CX3CR1 impairs the synaptic integration of adult‐born hippocampal granule neurons. Brain, Behavior, and Immunity, 68, 1–14. 10.1016/j.bbi.2017.10.002 29017970

[glia24090-bib-0009] Bridi, M. C. D. , Zong, F. J. , Min, X. , Luo, N. , Tran, T. , Qiu, J. , Severin, D. , Zhang, X. T. , Wang, G. , Zhu, Z. J. , He, K. W. , & Kirkwood, A. (2020). Daily oscillation of the excitation‐inhibition balance in visual cortical circuits. Neuron, 105(4), 621–629. 10.1016/j.neuron.2019.11.011 31831331 PMC9520672

[glia24090-bib-0010] Burkeen, J. F. , Womac, A. D. , Earnest, D. J. , & Zoran, M. J. (2011). Mitochondrial calcium signaling mediates rhythmic extracellular ATP accumulation in suprachiasmatic nucleus astrocytes. The Journal of Neuroscience, 31(23), 8432–8440. 10.1523/JNEUROSCI.6576-10.2011 21653847 PMC3125703

[glia24090-bib-0011] Chaudhury, D. , Wang, L. M. , & Colwell, C. S. (2005). Circadian regulation of hippocampal long‐term potentiation. Journal of Biological Rhythms, 20(3), 225–236. 10.1177/0748730405276352 15851529 PMC2581477

[glia24090-bib-0012] Choudhury, M. E. , Miyanishi, K. , Takeda, H. , Islam, A. , Matsuoka, N. , Kubo, M. , Matsumoto, S. , Kunieda, T. , Nomoto, M. , Yano, H. , & Tanaka, J. (2020). Phagocytic elimination of synapses by microglia during sleep. Glia, 68(1), 44–59. 10.1002/glia.23698 31429116

[glia24090-bib-0013] Dagher, N. N. , Najafi, A. R. , Kayala, K. M. , Elmore, M. R. , White, T. E. , Medeiros, R. , West, B. L. , & Green, K. N. (2015). Colony‐stimulating factor 1 receptor inhibition prevents microglial plaque association and improves cognition in 3xTg‐AD mice. Journal of Neuroinflammation, 12, 139. 10.1186/s12974-015-0366-9 PMC4522109 26232154

[glia24090-bib-0014] Dana, R. C. , & Martinez, J. L., Jr. (1984). Effect of adrenalectomy on the circadian rhythm of LTP. Brain Research, 308(2), 392–395. 10.1016/0006-8993(84)91086-2 6478217

[glia24090-bib-0015] de Vivo, L. , Bellesi, M. , Marshall, W. , Bushong, E. A. , Ellisman, M. H. , Tononi, G. , & Cirelli, C. (2017). Ultrastructural evidence for synaptic scaling across the wake/sleep cycle. Science, 355(6324), 507–510. 10.1126/science.aah5982 28154076 PMC5313037

[glia24090-bib-0017] Del Percio, C. , Drinkenburg, W. , Lopez, S. , Infarinato, F. , Bastlund, J. F. , Laursen, B. , Pedersen, J. T. , Christensen, D. Z. , Forloni, G. , Frasca, A. , Noè, F. M. , Bentivoglio, M. , Fabene, P. F. , Bertini, G. , Colavito, V. , Kelley, J. , Dix, S. , Richardson, J. C. , Babiloni, C. , … PharmaCog, C. (2017). On‐going electroencephalographic rhythms related to cortical arousal in wild‐type mice: The effect of aging. Neurobiology of Aging, 49, 20–30. 10.1016/j.neurobiolaging.2016.09.004 27728831

[glia24090-bib-0018] Durán, E. , Oyanedel, C. N. , Niethard, N. , Inostroza, M. , & Born, J. (2018). Sleep stage dynamics in neocortex and hippocampus. Sleep, 41(6), 1–11. 10.1093/sleep/zsy060 29893972

[glia24090-bib-0019] Dworak, M. , McCarley, R. W. , Kim, T. , Kalinchuk, A. V. , & Basheer, R. (2010). Sleep and brain energy levels: ATP changes during sleep. The Journal of Neuroscience, 30(26), 9007–9016. 10.1523/JNEUROSCI.1423-10.2010 20592221 PMC2917728

[glia24090-bib-0020] Elmore, M. R. , Najafi, A. R. , Koike, M. A. , Dagher, N. N. , Spangenberg, E. E. , Rice, R. A. , Kitazawa, M. , Matusow, B. , Nguyen, H. , West, B. L. , & Green, K. N. (2014). Colony‐stimulating factor 1 receptor signaling is necessary for microglia viability, unmasking a microglia progenitor cell in the adult brain. Neuron, 82(2), 380–397. 10.1016/j.neuron.2014.02.040 24742461 PMC4161285

[glia24090-bib-0021] Erblich, B. , Zhu, L. , Etgen, A. M. , Dobrenis, K. , & Pollard, J. W. (2011). Absence of colony stimulation factor‐1 receptor results in loss of microglia, disrupted brain development and olfactory deficits. PLoS One, 6(10), e26317. 10.1371/journal.pone.0026317 22046273 PMC3203114

[glia24090-bib-0022] Foley, J. , Blutstein, T. , Lee, S. , Erneux, C. , Halassa, M. M. , & Haydon, P. (2017). Astrocytic IP_3_/Ca^2+^ signaling modulates theta rhythm and REM sleep. Frontiers in Neural Circuits, 11, 3. 10.3389/fncir.2017.00003 PMC5253379 28167901

[glia24090-bib-0023] Fonken, L. K. , Frank, M. G. , Kitt, M. M. , Barrientos, R. M. , Watkins, L. R. , & Maier, S. F. (2015). Microglia inflammatory responses are controlled by an intrinsic circadian clock. Brain, Behavior, and Immunity, 45, 171–179. 10.1016/j.bbi.2014.11.009 25433170 PMC4386638

[glia24090-bib-0025] Garofalo, S. , Picard, K. , Limatola, C. , Nadjar, A. , Pascual, O. , & Tremblay, M. È. (2020). Role of glia in the regulation of sleep in health and disease. Comprehensive Physiology, 10(2), 687–712. 10.1002/cphy.c190022 32163207

[glia24090-bib-0026] Garofalo, S. , Porzia, A. , Mainiero, F. , Di Angelantonio, S. , Cortese, B. , Basilico, B. , Pagani, F. , Cignitti, G. , Chece, G. , Maggio, R. , Tremblay, M. E. , Savage, J. , Bisht, K. , Esposito, V. , Bernardini, G. , Seyfried, T. , Mieczkowski, J. , Stepniak, K. , Kaminska, B. , … Limatola, C. (2017). Environmental stimuli shape microglial plasticity in glioma. eLife, 6, 1–28. 10.7554/eLife.33415 PMC5774898 29286001

[glia24090-bib-0027] Ginhoux, F. , Greter, M. , Leboeuf, M. , Nandi, S. , See, P. , Gokhan, S. , Mehler, M. F. , Conway, S. J. , Ng, L. G. , Stanley, E. R. , Samokhvalov, I. M. , & Merad, M. (2010). Fate mapping analysis reveals that adult microglia derive from primitive macrophages. Science, 330(6005), 841–845. 20966214 10.1126/science.1194637 PMC3719181

[glia24090-bib-0028] Griffin, R. , Nally, R. , Nolan, Y. , McCartney, Y. , Linden, J. , & Lynch, M. A. (2006). The age‐related attenuation in long‐term potentiation is associated with microglial activation. Journal of Neurochemistry, 99(4), 1263–1272. 10.1111/j.1471-4159.2006.04165.x 16981890

[glia24090-bib-0029] Halassa, M. M. , Florian, C. , Fellin, T. , Munoz, J. R. , Lee, S.‐Y. , Abel, T. , Haydon, P. G. , & Frank, M. G. (2009). Astrocytic modulation of sleep homeostasis and cognitive consequences of sleep loss. Neuron, 61, 213–219. https://www.sciencedirect.com/science/article/pii/S0896627308010179 19186164 10.1016/j.neuron.2008.11.024 PMC2673052

[glia24090-bib-0030] Hall, S. , Deurveilher, S. , Robertson, G. S. , & Semba, K. (2020). Homeostatic state of microglia in a rat model of chronic sleep restriction. Sleep, 43(11), 1–16. https://academic.oup.com/sleep/article/43/11/zsaa108/5849344 10.1093/sleep/zsaa108 PMC7658640 32474610

[glia24090-bib-0031] Harris, K. M. , & Teyler, T. J. (1983). Age differences in a circadian influence on hippocampal LTP. Brain Research, 261(1), 69–73. 10.1016/0006-8993(83)91284-2 6301629

[glia24090-bib-0032] Hassani, O. K. , Lee, M. G. , & Jones, B. E. (2009). Melanin‐concentrating hormone neurons discharge in a reciprocal manner to orexin neurons across the sleep‐wake cycle. Proceedings of the National Academy of Sciences USA, 106, 2418–2422. 10.1073/pnas.0811400106 PMC2650171 19188611

[glia24090-bib-0033] Havekes, R. , & Aton, S. J. (2020). Impacts of sleep loss versus waking experience on brain plasticity: Parallel or orthogonal? Trends in Neurosciences, 43(6), 385–393. https://www.sciencedirect.com/science/article/pii/S0166223620300709 32459991 10.1016/j.tins.2020.03.010 PMC7505037

[glia24090-bib-0035] Hide, I. , Tanaka, M. , Inoue, A. , Nakajima, K. , Kohsaka, S. , Inoue, K. , & Nakata, Y. (2000). Extracellular atp triggers tumor necrosis factor‐α release from rat microglia. Journal of Neurochemistry, 75, 965–972. 10.1046/j.1471-4159.2000.0750965.x 10936177

[glia24090-bib-0036] Huang, Y. , Xu, Z. , Xiong, S. , Sun, F. , Qin, G. , Hu, G. , Wang, J. , Zhao, L. , Liang, Y. X. , & Wu, T. (2018). Repopulated microglia are solely derived from the proliferation of residual microglia after acute depletion. Nature Neuroscience, 21(4), 530–540. https://www.nature.com/articles/s41593-018-0090-8 29472620 10.1038/s41593-018-0090-8

[glia24090-bib-0038] Ingiosi, A. M. , Opp, M. R. , & Krueger, J. M. (2013). Sleep and immune function: Glial contributions and consequences of aging. Current Opinion in Neurobiology, 23(5), 806–811. 10.1016/j.conb.2013.02.003 23452941 PMC3695049

[glia24090-bib-0039] Jilg, A. J. , Bechstein, P. , Saade, A. , Dick, M. , Li, T. X. , Tosini, G. , Rami, A. , Zemmar, A. , & Stehle, J. H. (2019). Melatonin modulates daytime‐dependent synaptic plasticity and learning efficiency. Journal of Pineal Research, 66(3), e12553. 10.1111/jpi.12553 30618149 PMC6405292

[glia24090-bib-0040] Jin, W. N. , Shi, S. X. , Li, Z. , Li, M. , Wood, K. , Gonzales, R. J. , & Liu, Q. (2017). Depletion of microglia exacerbates postischemic inflammation and brain injury. Journal of Cerebral Blood Flow and Metabolism, 37(6), 2224–2236. 10.1177/0271678X17694185 28273719 PMC5444553

[glia24090-bib-0041] Jung, S. , Aliberti, J. , Graemmel, P. , Sunshine, M. J. , Kreutzberg, G. W. , Sher, A. , & Littman, D. R. (2000). Analysis of fractalkine receptor CX(3)CR1 function by targeted deletion and green fluorescent protein reporter gene insertion. Molecular and Cellular Biology, 20(11), 4106–4114. 10.1128/mcb.20.11.4106-4114.2000 10805752 PMC85780

[glia24090-bib-0042] Kana, V. , Desland, F. A. , Casanova‐Acebes, M. , Ayata, P. , Badimon, A. , Nabel, E. , Yamamuro, K. , Sneeboer, M. , Tan, I.‐L. , & Flanigan, M. E. (2019). CSF‐1 controls cerebellar microglia and is required for motor function and social interaction. The Journal of Experimental Medicine, 216(10), 2265–2281. https://rupress.org/jem/article/216/10/2265/120545 31350310 10.1084/jem.20182037 PMC6781012

[glia24090-bib-0043] Klinzing, J. G. , Niethard, N. , & Born, J. (2019). Mechanisms of systems memory consolidation during sleep. Nature Neuroscience, 22(10), 1598–1610. 10.1038/s41593-019-0467-3 31451802

[glia24090-bib-0044] Krueger, J. M. , Taishi, P. , De, A. , Davis, C. J. , Winters, B. D. , Clinton, J. , Szentirmai, É. , & Zielinski, M. R. (2010). ATP and the purine type 2 X7 receptor affect sleep. Journal of Applied Physiology, 109, 1318–1327. https://journals.physiology.org/doi/full/10.1152/japplphysiol.00586.2010 20829501 10.1152/japplphysiol.00586.2010 PMC2980381

[glia24090-bib-0046] Lauro, C. , Cipriani, R. , Catalano, M. , Trettel, F. , Chece, G. , Brusadin, V. , Antonilli, L. , van Rooijen, N. , Eusebi, F. , Fredholm, B. B. , & Limatola, C. (2010). Adenosine A1 receptors and microglial cells mediate CX3CL1‐induced protection of hippocampal neurons against Glu‐induced death. Neuropsychopharmacology, 35(7), 1550–1559. 10.1038/npp.2010.26 20200508 PMC3055460

[glia24090-bib-0047] Lee, M. G. , Hassani, O. K. , & Jones, B. E. (2005). Discharge of identified orexin/hypocretin neurons across the sleep‐waking cycle. Journal of Neuroscience, 25, 6716–6720. 10.1523/JNEUROSCI.1887-05.2005 16014733 PMC6725432

[glia24090-bib-0048] Lei, F. , Cui, N. , Zhou, C. , Chodosh, J. , Vavvas, D. G. , & Paschalis, E. I. (2020). CSF1R inhibition by a small‐molecule inhibitor is not microglia specific; affecting hematopoiesis and the function of macrophages. Proceedings of the National Academy of Science USA, 117(38), 23336–23338. 10.1073/pnas.1922788117 PMC7519218 32900927

[glia24090-bib-0049] Leung, L. S. , Shen, B. , Rajakumar, N. , & Ma, J. (2003). Cholinergic activity enhances hippocampal long‐term potentiation in CA1 during walking in rats. The Journal of Neuroscience, 23(28), 9297–9304. 10.1523/JNEUROSCI.23-28-09297.2003 14561856 PMC6740561

[glia24090-bib-0050] Limatola, C. , & Ransohoff, R. M. (2014). Modulating neurotoxicity through CX3CL1/CX3CR1 signaling. Frontiers in Cellular Neuroscience, 8, 1–8. 10.3389/fncel.2014.00229 25152714 PMC4126442

[glia24090-bib-0051] Liu, Y. J. , Spangenberg, E. E. , Tang, B. , Holmes, T. C. , Green, K. N. , & Xu, X. (2021). Microglia elimination increases neural circuit connectivity and activity in adult mouse cortex. The Journal of Neuroscience, 41(6), 1274–1287. 10.1523/JNEUROSCI.2140-20.2020 33380470 PMC7888230

[glia24090-bib-0052] Liu, Y. U. , Ying, Y. , Li, Y. , Eyo, U. B. , Chen, T. , Zheng, J. , Umpierre, A. D. , Zhu, J. , Bosco, D. B. , Dong, H. , & Wu, L. J. (2019). Neuronal network activity controls microglial process surveillance in awake mice via norepinephrine signaling. Nature Neuroscience, 22(11), 1771–1781. 10.1038/s41593-019-0511-3 31636449 PMC6858573

[glia24090-bib-0053] Liu, Z. W. , Faraguna, U. , Cirelli, C. , Tononi, G. , & Gao, X. B. (2010). Direct evidence for wake‐related increases and sleep‐related decreases in synaptic strength in rodent cortex. The Journal of Neuroscience, 30(25), 8671–8675. 10.1523/JNEUROSCI.1409-10.2010 20573912 PMC2903226

[glia24090-bib-0054] Maggi, L. , Scianni, M. , Branchi, I. , D'Andrea, I. , Lauro, C. , & Limatola, C. (2011). CX(3)CR1 deficiency alters hippocampal‐dependent plasticity phenomena blunting the effects of enriched environment. Frontiers in Cellular Neuroscience, 5, 22. 10.3389/fncel.2011.00022 PMC3198035 22025910

[glia24090-bib-0056] Maret, S. , Faraguna, U. , Nelson, A. B. , Cirelli, C. , & Tononi, G. (2011). Sleep and waking modulate spine turnover in the adolescent mouse cortex. Nature Neuroscience, 14(11), 1418–1420. 10.1038/nn.2934 21983682 PMC3203346

[glia24090-bib-0057] Marpegan, L. , Swanstrom, A. E. , Chung, K. , Simon, T. , Haydon, P. G. , Khan, S. K. , Liu, A. C. , Herzog, E. D. , & Beaulé, C. (2011). Circadian regulation of ATP release in astrocytes. The Journal of Neuroscience, 31(23), 8342–8350. 10.1523/JNEUROSCI.6537-10.2011 21653839 PMC3135876

[glia24090-bib-0058] Meerlo, P. , Koehl, M. , van der Borght, K. , & Turek, F. W. (2002). Sleep restriction alters the hypothalamic‐pituitary‐adrenal response to stress. Journal of Neuroendocrinology, 14(5), 397–402. 10.1046/j.0007-1331.2002.00790.x 12000545

[glia24090-bib-0059] Milior, G. , Lecours, C. , Samson, L. , Bisht, K. , Poggini, S. , Pagani, F. , Deflorio, C. , Lauro, C. , Alboni, S. , Limatola, C. , Branchi, I. , Tremblay, M. È. , & Maggi, L. (2016). Fractalkine receptor deficiency impairs microglial and neuronal responsiveness to chronic stress. Brain, Behavior, and Immunity, 55, 114–125. 10.1016/j.bbi.2015.07.024 26231972

[glia24090-bib-0060] Nadjar, A. , Blutstein, T. , Aubert, A. , Laye, S. , & Haydon, P. G. (2013). Astrocyte‐derived adenosine modulates increased sleep pressure during inflammatory response. Glia, 61(5), 724–731. 10.1002/glia.22465 23378051

[glia24090-bib-0061] Niethard, N. , Burgalossi, A. , & Born, J. (2017). Plasticity during sleep is linked to specific regulation of cortical circuit activity. Frontiers in Neural Circuits, 11, 65. 10.3389/fncir.2017.00065 PMC5605564 28966578

[glia24090-bib-0063] Ono, D. , & Yamanaka, A. (2017). Hypothalamic regulation of the sleep/wake cycle. Neuroscience Research, 118, 74–81. 10.1016/j.neures.2017.03.013 28526553

[glia24090-bib-0065] Pankratov, Y. , Ulyana Lalo, U. , Verkhratsky, A. , & North, R. A. (2006). Vesicular release of ATP at central synapses. Pflügers Archiv, 452, 589–597. 16639550 10.1007/s00424-006-0061-x

[glia24090-bib-0066] Paolicelli, R. C. , Bolasco, G. , Pagani, F. , Maggi, L. , Scianni, M. , Panzanelli, P. , Giustetto, M. , Ferreira, T. A. , Guiducci, E. , Dumas, L. , Ragozzino, D. , & Gross, C. (2011). Synaptic pruning by microglia is necessary for normal brain development. Science, 333, 1456–1458. 10.1126/science.1202529 21778362

[glia24090-bib-0067] Paolicelli, R. C. , Bisht, K. , & Tremblay, M. È. (2014). Fractalkine regulation of microglial physiology and consequences on the brain and behavior. Frontiers in Cellular Neuroscience, 8, 129. 10.3389/fncel.2014.00129 PMC4026677 24860431

[glia24090-bib-0068] Prieto, G. A. , Tong, L. , Smith, E. D. , & Cotman, C. W. (2019). TNFα and IL‐1β but not IL‐18 suppresses hippocampal long‐term potentiation directly at the synapse. Neurochemistry Research, 44, 49–60. 10.1007/s11064-018-2517-8 PMC6170724 29619614

[glia24090-bib-0069] Porkka‐Heiskanen, T. , Strecker, R. E. , Thakkar, M. , Bjorkum, A. A. , Greene, R. W. , & McCarley, R. W. (1997). Adenosine: A mediator of the sleep‐inducing effects of prolonged wakefulness. Science, 276(5316), 1265–1268. 10.1126/science.276.5316.1265 9157887 PMC3599777

[glia24090-bib-0070] Raghavan, A. V. , Horowitz, J. M. , & Fuller, C. A. (1999). Diurnal modulation of long‐term potentiation in the hamster hippocampal slice. Brain Research, 833(2), 311–314. 10.1016/s0006-8993(99)01523-1 10375711

[glia24090-bib-0071] Rodrigues, R. J. , Tomé, A. R. , & Cunha, R. A. (2015). ATP as a multi‐target danger signal in the brain. Frontiers in Neuroscience, 9, 148. 10.3389/fnins.2015.00148 PMC4412015 25972780

[glia24090-bib-0073] Schmittgen, T. D. , & Livak, K. J. (2008). Analyzing real‐time PCR data by the comparative C(T) method. Nature Protocols, 3(6), 1101–1108. 10.1038/nprot.2008.73 18546601

[glia24090-bib-0074] Shieh, C. H. , Heinrich, A. , Serchov, T. , van Calker, D. , & Biber, K. (2014). P2X7‐dependent, but differentially regulated release of IL‐6, CCL2, and TNF‐α in cultured mouse microglia. Glia, 62, 592–607. 10.1002/glia.22628 24470356

[glia24090-bib-0075] Stanhope, B. A. , Jaggard, J. B. , Gratton, M. , Brown, E. B. , & Keene, A. C. (2020). Sleep regulates glial plasticity and expression of the engulfment receptor draper following neural injury. Current Biology, 30(6), 1092–1101. 10.1016/j.cub.2020.02.057 32142708

[glia24090-bib-0076] Stenberg, D. , Litonius, E. , Halldner, L. , Johansson, B. , Fredholm, B. B. , & Porkka‐Heiskanen, T. (2003). Sleep and its homeostatic regulation in mice lacking the adenosine A1 receptor. Journal of Sleep Research, 12(4), 283–290. 10.1046/j.0962-1105.2003.00367.x 14633239

[glia24090-bib-0077] Stowell, R. D. , Sipe, G. O. , Dawes, R. P. , Batchelor, H. N. , Lordy, K. A. , Whitelaw, B. S. , Stoessel, M. B. , Bidlack, J. M. , Brown, E. , Sur, M. , & Majewska, A. K. (2019). Noradrenergic signaling in the wakeful state inhibits microglial surveillance and synaptic plasticity in the mouse visual cortex. Nature Neuroscience, 22(11), 1782–1792. 10.1038/s41593-019-0514-0 31636451 PMC6875777

[glia24090-bib-0078] Tononi, G. , & Cirelli, C. (2003). Sleep and synaptic homeostasis: A hypothesis. Brain Research Bulletin, 62(2), 143–150. 10.1016/j.brainresbull.2003.09.004 14638388

[glia24090-bib-0080] Trettel, F. , Di Castro, M. A. , & Limatola, C. (2020). Chemokines: Key molecules that orchestrate communication among neurons, microglia and astrocytes to preserve brain function. Neuroscience, 439, 230–240. 10.1016/j.neuroscience.2019.07.035 31376422

[glia24090-bib-0081] Tuan, L. H. , & Lee, L. J. (2019). Microglia‐mediated synaptic pruning is impaired in sleep‐deprived adolescent mice. Neurobiology of Disease, 130, 104517. 10.1016/j.nbd.2019.104517 31229687

[glia24090-bib-0082] Vyazovskiy, V. V. , Cirelli, C. , Pfister‐Genskow, M. , Faraguna, U. , & Tononi, G. (2008). Molecular and electrophysiological evidence for net synaptic potentiation in wake and depression in sleep. Nature Neuroscience, 11(2), 200–208. 10.1038/nn2035 18204445

[glia24090-bib-0083] Wadhwa, M. , Kumari, P. , Chauhan, G. , Roy, K. , Alam, S. , Kishore, K. , Ray, K. , & Panjwani, U. (2017). Sleep deprivation induces spatial memory impairment by altered hippocampus neuroinflammatory responses and glial cells activation in rats. Journal of Neuroimmunology, 312, 38–48. 10.1016/j.jneuroim.2017.09.003 28912034

[glia24090-bib-0086] Wisor, J. P. , Schmidt, M. A. , & Clegern, W. C. (2011). Evidence for neuroinflammatory and microglial changes in the cerebral response to sleep loss. Sleep, 34(3), 261–272. 10.1093/sleep/34.3.261 21358843 PMC3041702

[glia24090-bib-0087] Wynne, A. M. , Henry, C. J. , Huang, Y. , Cleland, A. , & Godbout, J. P. (2010). Protracted downregulation of CX3CR1 on microglia of aged mice after lipopolysaccharide challenge. Brain, Behavior, and Immunity, 24(7), 1190–1201. 10.1016/j.bbi.2010.05.011 20570721 PMC2939290

[glia24090-bib-0088] Xie, L. , Kang, H. , Xu, Q. , Chen, M. J. , Liao, Y. , Thiyagarajan, M. , O'Donnell, J. , Christensen, D. J. , Nicholson, C. , Iliff, J. J. , Takano, T. , Deane, R. , & Nedergaard, M. (2013). Sleep drives metabolite clearance from the adult brain. Science, 342(6156), 373–377. 10.1126/science.1241224 24136970 PMC3880190

[glia24090-bib-0089] Yang, G. , Lai, C. S. , Cichon, J. , Ma, L. , Li, W. , & Gan, W. B. (2014). Sleep promotes branch‐specific formation of dendritic spines after learning. Science, 344(6188), 1173–1178. 10.1126/science.1249098 24904169 PMC4447313

[glia24090-bib-0090] Yegla, B. , Boles, J. , Kumar, A. , & Foster, T. C. (2021). Partial microglial depletion is associated with impaired hippocampal synaptic and cognitive function in young and aged rats. Glia, 69, 1494–1514. 10.1002/glia.23975 33586813 PMC8278544

[glia24090-bib-0091] Zhan, Y. , Paolicelli, R. C. , Sforazzini, F. , Weinhard, L. , Bolasco, G. , Pagani, F. , Vyssotski, A. L. , Bifone, A. , Gozzi, A. , Ragozzino, D. , & Gross, C. T. (2014). Deficient neuron‐microglia signaling results in impaired functional brain connectivity and social behavior. Nature Neuroscience, 17(3), 400–406. 10.1038/nn.3641 24487234

[glia24090-bib-0092] Zhou, Y. , Lai, C. S. W. , Bai, Y. , Li, W. , Zhao, R. , & Yang, G. (2020). REM sleep promotes experience‐dependent dendritic spine elimination in the mouse cortex. Nature Communications, 11, 4819. 10.1038/s41467-020-18592-5 PMC7511313 32968048

[glia24090-bib-0093] Zucker, R. S. (1989). Short‐term synaptic plasticity. Annual Review of Neuroscience, 12, 13–31. 10.1146/annurev.ne.12.030189.000305 2648947

