# Supplementary material for: Microglia modulate hippocampal synaptic transmission and sleep duration along the light/dark cycle
Source: Glia. 2021 Sep 6;70(1):89–105. doi: 10.1002/glia.24090 (PMC9291950; doi:10.1002/glia.24090)
Supplement: Supplementary file 1 — Appendix S1. Supporting Information. [file GLIA-70-89-s001.docx]

**Supplementary information**

**Methods**

**Immunostaining and image acquisition for microglial depletion validation.** Mice treated with PLX5622 or control chow for 7 days were anesthetized and intracardially perfused with phosphate buffered saline solution (PBS, Sigma) and 4% paraformaldehyde (PFA, Santa Cruz Biotechnology); brains were then isolated, fixed in PFA and snap frozen. Cryostat sections (20 μm) were washed in PBS, blocked (3% goat serum and 0.3% Triton X-100) for 1 h at room temperature and incubated overnight at 4° C with rabbit anti-IBA1 (Wako Cat# 019-19741, RRID: AB_839504) diluted in PBS (1:500) containing 1% goat serum and 0.1% Triton X-100. After several washes, sections were stained with the fluorophore-conjugated antibody and Hoechst for nuclei visualization. Fluorescence images were digitized using a CoolSNAP camera (Photometrics) coupled to an ECLIPSE Ti-S microscope (Nikon) and processed using the MetaMorph 7.6.5.0 software (Molecular Device). Slices were scanned by consecutive fields of vision (x10) to build a single image per section. Data are expressed as the percentage of reduction of the area occupied by fluorescent cells in PLX5622-treated mice versus control. For comparison between different treatments, at least 9 coronal sections from 3 mice per condition were analysed.

**Motor activity recordings.** Digital individually ventilated caging system (DVC™) is a home cage monitoring device manufactured by Tecniplast SpA (Buguggiate, Italy), with a sensing board installed underneath each cage. The sensing board is suited with 12 electrodes connected to an integrated circuit that continuously measures their electrical capacitance. Mice movements induce significant capacitance changes while close to electrodes, and thus, by properly tracking these changes over time, the DVC monitors animal activity in the cage (Iannello et al., 2019). Capacitance remains substantially unchanged when material compositions around an electrode is unvaried. Each sensing board is connected to a dedicated IT workstation, which provides power and data connection. No cables and sensors are connected directly to the cage, which is untouched by the digitalization of the rack (all sensing occurs externally to the cage and non-intrusively). For this study, dedicated IT unit collected raw data from each electrode of cage 4 times per second (i.e., 4 Hz).

**Circadian metrics analysis in light:dark condition.** Microglia depleted (n = 15) and age-matched control (n = 18) mice were housed in the DVC tracking system to monitor their spontaneous motor activity. PLX5622 treatment and control chow administration started 7 days before the housing in the DVC and continued until the end of the experiment. Animals were allowed to acclimatize to the system for 3 days, then we analysed the following circadian metrics for 5 consecutive days: (i) average hourly activity; (ii) averaged phase activity for ZT 0-12 and ZT 12-0; (iii) diurnality, intended as the percentage between the activity between in ZT 0-12 and the activity in ZT 0-0. Acrophase is the time at which the peak of the circadian rhythm occurs and is estimated by using Cosinor Analysis (2). This method is based on fitting a cosine curve with defined period (T=24 hours) by least squares to each daily minute activity time series. The acrophase is then determined as the time at which the fitted wave reaches its maximum.

**Circadian metrics analysis in dark:dark condition.**Mice were housed in the DVC® tracking system in a dedicate dark room. PLX5622 treatment and control chow administration started 7 days after the housing in the DVC and continued until the end of the experiment (day 26). Considering that in dark:dark condition external stimuli are absent, time is expressed as circadian time (CT; i. e., CT 7 = 7 am). We analysed the following circadian metrics from day 22 to day 26 (to ensure microglia depletion): (i) average hourly activity; (ii) averaged phase activity for CT 7-19 and CT 19-7; (iii) phase shift, intended as the average daily shift of the activity onset. We estimated activity onset (data not shown) by a template-matching algorithm, used by the ClockLab analysis package (Actimetrics Inc.) and adapted to DVC spontaneous locomotion data in (Fuochi et al., 2021). The daily phase shift was determined as the coefficient of linear regression of the onset data against time (Refinetti, 2007). Diurnality is intended as the percentage between the activity between in CT 7-19 and the activity in CT 7-7. For this experiment, we used 6 control mice and 5 PLX5622-treated mice.

**EEG/EMG data recording.** To confirm and investigate sleep-wake cycle and sleep quality using another method of microglial depletion, we performed 24 h EEG/EMG recordings in mice treated with either PLX3397 (n = 6) or the vehicle (n = 5).
Mice were anaesthetized with isoflurane (4% for induction, 1–2% for maintenance) and a subcutaneous injection of buprenorphine (0.05 mg/kg), then implanted with head mounts (Pinnacle Technologies, Lawrence, Kansas, USA) to record the EEG and EMG activities. Stainless steel screws were aligned centrally along the sagittal suture: two were placed anterior to bregma and two were placed posterior to bregma. EMG electrodes were inserted into the neck muscles and the implant was secured to the skull using dental acrylic (Lang Dental Manufacturing Co) (Decoeur et al., 2020; Nadjar et al., 2013). One week post-surgery, mice were connected to the recording device for 3 additional days of habituation and their EEG-EMG data were recorded on the fourth day over 24 h (starting at 8:00 a.m., at ZT0). Mice were then unplugged from the system and received oral gavage of either PLX3397 or the vehicle solution for 21 days. After the 21 days, mice were reconnected to the system for 48 h of habituation followed by 24 h of recording.
EEG and EMG signals were recorded continuously using the 8400-recording system (Pinnacle Technology Inc). The EEG/EMG signals were analysed in 4-sec periods semi-automatically by an experimenter blinded to the groups using the SleepSign software (KISSEI COMTEC CO., LTD, Matsumoto, Japan), as previously described (Decoeur et al., 2020; Nadjar et al., 2013). Based on the

level of EEG and EMG activities, we defined 3 vigilant states, including wakefulness and 2 sleep stages: NREM and REM. Data were analysed as a percentage of total recording time over 12 h and 24 h after stage scoring. The sleep pressure was quantified using the amplitude of delta oscillations (between 0.5 and 4 Hz) in the light phase (Decoeur et al., 2020, Halassa et al., 2009).
For the EEG experiments (2) with PLX3397, statistical analysis was conducted using Two-way ANOVA (repeated measures) with the software Prism (GraphPad, Version 9). Post-hoc comparisons were performed using Bonferroni’s multiple comparisons test and mean differences were considered statistically significant when p < 0.05.

**Field Excitatory Post Synaptic Potential (fEPSP) recordings for LTP protocol.** After cutting, the slices were transferred to the recording chamber interface (BSC1, Scientific System Design Inc), maintained at 30-32°C and constantly superfused at the rate of 2.5 ml/min with oxygenated ACSF. Stimulus intensity was adjusted to evoke fEPSP of amplitude about 50% of the maximal amplitude with minimal contamination by a population spike. Evoked responses were monitored online and stable baseline responses were recorded for at least 10 min. Only the slices that showed stable fEPSP amplitudes were included in the experiments. LTP was induced by high-frequency stimulation (HFS, 1 train of stimuli at 100 Hz of 1 s duration), repeated after 30 min. To analyse the time-course of the fEPSP amplitude, the recorded fEPSP was routinely averaged over 1 min (n = 3). The fEPSP amplitude changes following the LTP induction protocol were calculated at 20 min post HFS with respect to the baseline. N/n refers to the number of slices on the total number of mice analysed.

**Table S1.**

| **(A) Within groups** | |  |  |  |  |  |  |  |
| --- | --- | --- | --- | --- | --- | --- | --- | --- |
| **Groups** | **Phases** | **Test** | **Variables** | **T** | **Z** | **P** | | **Effect size** |
| C57BL/6 | Light vs Dark | Wilcoxon | Total sleep | 0.0 | 2.80 | 0.003 (*) | | 2.36 |
|  |  |  | NREM | 0.0 | 2.80 | 0.003 (*) | | 2.34 |
|  |  |  | REM | 6.0 | 2.19 | 0.014 | | 0.40 |
|  |  |  | Total wakefulness | 0.0 | 2.80 | 0.003 (*) | | -2.36 |
|  |  |  | Movement | 0.0 | 2.80 | 0.003 (*) | | -2.48 |
|  |  |  | Passive Wake | 24.0 | 0.36 | 0.361 | | -0.12 |
| PLX5622 | Light vs Dark | Wilcoxon | Total sleep | 1.0 | 2.55 | 0.005 (*) | | 1.56 |
|  |  |  | NREM | 1.0 | 2.55 | 0.005 (*) | | 1.64 |
|  |  |  | REM | 14.5 | 0.49 | 0.312 | | 0.27 |
|  |  |  | Total wakefulness | 1.0 | 2.57 | 0.005 (*) | | -1.56 |
|  |  |  | Movement | 0.0 | 2.66 | 0.004 (*) | | -2.47 |
|  |  |  | Passive Wake | 2.0 | 2.43 | 0.008 | | 1.03 |
| C57BL/6 J | Light vs Dark | Wilcoxon | Total sleep | 0.0 | 1.83 | 0.034 | | 5.82 |
|  |  |  | NREM | 0.0 | 1.83 | 0.034 | | 5.80 |
|  |  |  | REM | 0.0 | 1.60 | 0.054 | | 1.16 |
|  |  |  | Total wakefulness | 0.0 | 1.83 | 0.034 | | -5.82 |
|  |  |  | Movement | 1.0 | 1.46 | 0.072 | | -1.81 |
|  |  |  | Passive Wake | 0.0 | 1.83 | 0.034 | | -2.77 |
| *cx3cr1*  *^GFP/GFP^* | Light vs Dark | Wilcoxon | Total sleep | 0.00 | 2.37 | 0.009 | | 2.36 |
|  |  |  | NREM | 0.00 | 2.37 | 0.009 | | 2.85 |
|  |  |  | REM | 1.00 | 1.46 | 0.072 | | 0.63 |
|  |  |  | Total wakefulness | 0.00 | 2.37 | 0.009 | | -2.36 |
|  |  |  | Movement | 0.00 | 2.37 | 0.009 | | -2.45 |
|  |  |  | Passive Wake | 9.00 | 0.85 | 0.199 | | -0.42 |
| **(B) Between groups** | |  |  |  |  |  |  |  |
| **Groups** | **Phases** | **Test** | **Variable** | **U** | **Z** | P | | Effect size |
| C57BL/6 vs PLX5622 | Light | Mann-Whitney | Total sleep | 41.00 | -0.29 | 0.388 | | -0.13 |
|  |  |  | NREM | 41.00 | -0.29 | 0.388 | | -0.10 |
|  |  |  | REM | 39.00 | -0.45 | 0.327 | | -0.20 |
|  |  |  | Total wakefulness | 41.00 | 0.29 | 0.388 | | 0.13 |
|  |  |  | Movement | 31.00 | 1.10 | 0.135 | | 0.36 |
|  |  |  | Passive Wake | 37.00 | -0.61 | 0.270 | | -0.21 |
| C57BL/6 vs PLX5622 | Dark | Mann-Whitney | Total sleep | 10.00 | -2.82 | 0.002 (*) | | -1.61 |
|  |  |  | NREM | 11.00 | -2.74 | 0.003 (*) | | -1.60 |
|  |  |  | REM | 29.50 | -1.22 | 0.110 | | -0.42 |
|  |  |  | Total wakefulness | 10.00 | 2.82 | 0.002 (*) | | 1.61 |
|  |  |  | Movement | 26.00 | 1.51 | 0.065 | | 1.12 |
|  |  |  | Passive Wake | 32.00 | 1.02 | 0.154 | | 0.75 |
| C57BL/6 J vs *cx3cr1*  *^GFP/GFP^* | Light | Mann-Whitney | Total sleep | 13.00 | 0.09 | | 0.462 | 0.05 |
|  |  |  | NREM | 13.00 | 0.09 | | 0.462 | 0.03 |
|  |  |  | REM | 9.50 | 0.76 | | 0.225 | 0.29 |
|  |  |  | Total wakefulness | 13.00 | -0.09 | | 0.462 | - 0.05 |
|  |  |  | Movement | 8.00 | 1.04 | | 0.149 | 0.89 |
|  |  |  | Passive Wake | 4.00 | -1.80 | | 0.036 | -1.77 |
| C57BL/6 J vs *cx3cr1*  *^GFP/GFP^* | Dark | Mann-Whitney | Total sleep | 4.00 | -1.80 | | 0.036 | -1.46 |
|  |  |  | NREM | 4.00 | -1.80 | | 0.036 | -1.46 |
|  |  |  | REM | 12.50 | -0.19 | | 0.425 | -0.30 |
|  |  |  | Total wakefulness | 4.00 | 1.80 | | 0.036 | 1.46 |
|  |  |  | Movement | 14.00 | 0.00 | | 0.500 | 0.03 |
|  |  |  | Passive Wake | 6.00 | 1.42 | | 0.078 | 1.52 |

**Table S1. Statistical analysis of total sleep, NREM, REM, total wakefulness, movement and passive wake in the different experimental groups.**

**(A)** Within-group statistical analysis of total sleep (i.e., NREM+REM), NREM, REM, total wakefulness (movement + passive wake), movement and passive wake between the light and dark phases in PLX5622-treated mice, *cx3cr1^GFP/GFP^* and control mice (C57BL/6N and C57BL/6J respectively). Non-parametric Wilcoxon test was applied due to the limited sample size. **(B)** Between-group statistical analysis for the different conditions. Non-parametric Mann-Whitney U test was used due to the limited sample size. Bonferroni-corrected statistically significant p values are reported in bold (p < 0.05/8 = 0.006 = p < 0.05 corrected).

**Table S2.**

| **Conditions** | | **C57BL/6**  **(N = 10)**  *Mean ± s.e.m (h)* | **PLX5622**  **(N = 9)**  *Mean ± s.e.m (h)* | **C57BL/6J**  **(N = 4)**  *Mean ± s.e.m (h)* | **CX3CR1**  **(N = 7)**  *Mean ± s.e.m (h)* |
| --- | --- | --- | --- | --- | --- |
| **Sleep onset** | Light | 0.29 ± 0.07 | 0.12 ± 0.05 (*) | 0.09 ± 0.09 | 0.26 ± 0.1 |
|  | Dark | 0.89 ± 0.4 | 0.17 ± 0.07 (*) | 0.85 ± 0.6 | 0.37 ± 0.1 |

**Table S2. Mean values and statistics of sleep onset after light changes in the different conditions.**

Mean ± standard error of the mean (s.e.m.) of the hours (h) between the light change and the sleep onset (i.e., the moment when the mice fall asleep) in PLX5622-treated mice, *cx3cr1^GFP/GFP^* and control mice (C57BL/6N and C57BL/6J respectively). The sleep onset was calculated for the light and dark phases. * p < 0.05 uncorrected.

**Table S3.**

| **(A) Within groups** | |  |  |  |  |  |  |
| --- | --- | --- | --- | --- | --- | --- | --- |
| **Groups** | **Phases** | **Test** | **Variable** | **T** | **Z** | **P** | **Effect**  **size** |
| C57BL/6 | Light vs Dark | Wilcoxon | Sleep onset | 13.00 | 1.48 | 0.070 | -0.72 |
| PLX5622 | Light vs Dark | Wilcoxon | Sleep onset | 18.00 | 0.53 | 0.297 | -0.28 |
| C57BL/6J | Light vs Dark | Wilcoxon | Sleep onset | 2.00 | 1.09 | 0.137 | -1.02 |
| *cx3cr1^GFP/GFP^* | Light vs Dark | Wilcoxon | Sleep onset | 8.00 | 0.92 | 0.15 | -0.38 |
| **(B) Between groups** | |  |  |  |  |  |  |
| **Groups** | **Phases** | **Test** | **Variable** | **U** | **Z** | **P** | **Effect**  **size** |
| C57BL/6 vs PLX5622 | Light | Mann-Whitney | Sleep onset | 23.00 | 1.75 | 0.040 (*) | 0.94 |
| C57BL/6 vs PLX5622 | Dark | Mann-Whitney | Sleep onset | 18.00 | 2.16 | 0.015 (*) | 0.85 |
| C57BL/6J vs *cx3cr1^GFP/GFP^* | Light | Mann-Whitney | Sleep onset | 6.50 | -1.32 | 0.093 | -0.70 |
| C57BL/6J vs *cx3cr1^GFP/GFP^* | Dark | Mann-Whitney | Sleep onset | 14.00 | 0.00 | 0.500 | 0.73 |

**Table S3.  Statistical analysis of the latency of sleep onset in the experimental groups.**

**(A)** Within-group statistical comparison of the latency of sleep onset in PLX5622-treated mice, cx3cr1GFP/GFP and control mice (C57BL/6N and C57BL/6J respectively). Non-parametric Wilcoxon test was used due to the limited sample size. **(B)** For between-group statistical comparison the Mann-Whitney U test was used. For exploratory purposes, we did not use the Bonferroni correction. * p < 0.05 uncorrected.
