# Supplementary figures and images for: Microglia modulate hippocampal synaptic transmission and sleep duration along the light/dark cycle
Source: Glia. 2021 Sep 6;70(1):89–105. doi: 10.1002/glia.24090 (PMC9291950; doi:10.1002/glia.24090)

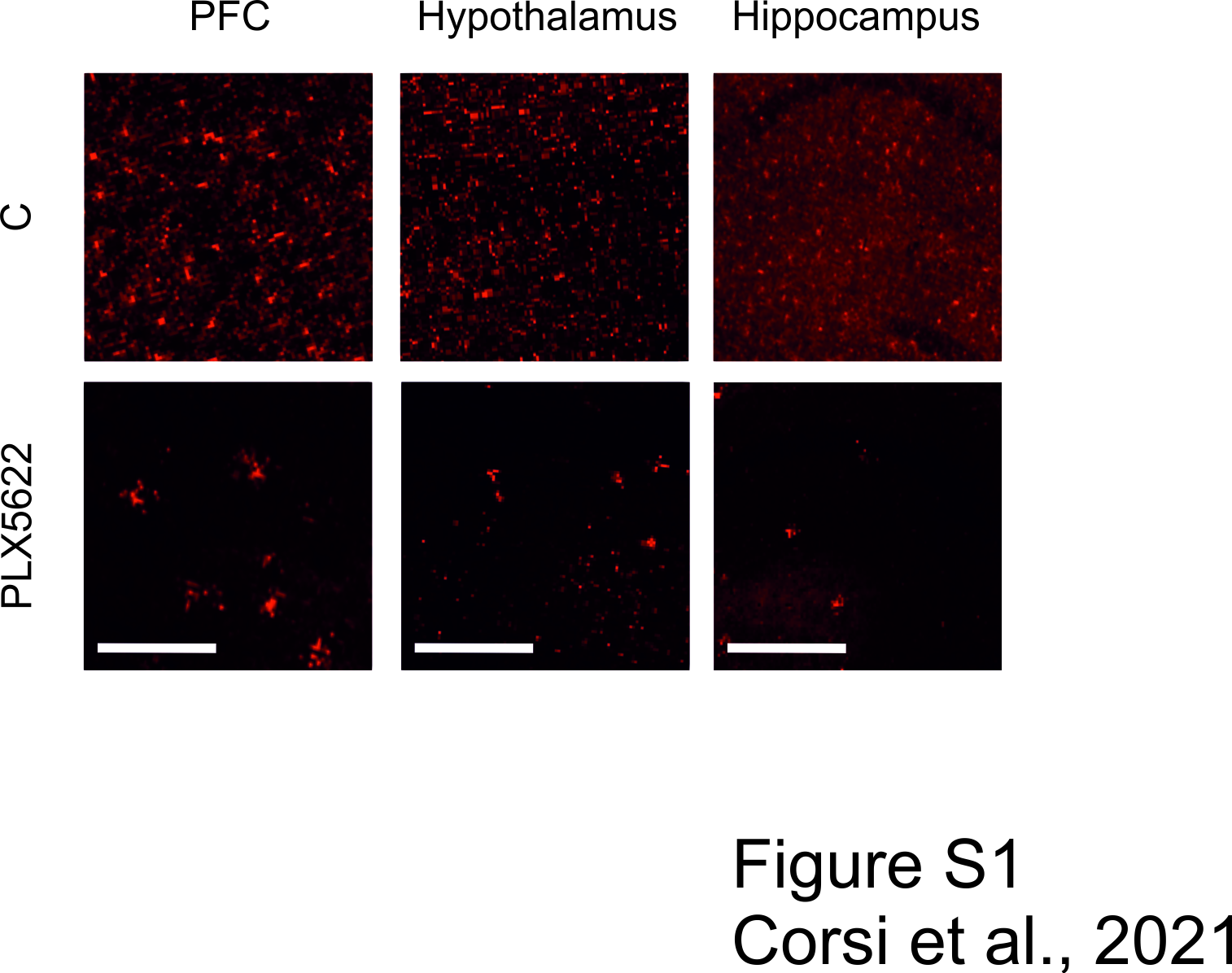

Supplement: Supplementary file 2 — Figure S1 Effectiveness of PLX5622 treatment in depleting microglia. Representative images showing IBA1‐stained cells in PFC, hypothalamus and hippocampus for controls (upper panels) and mice treated with PLX5622 for at least 7 days (lower panels). Scale bars: PFC 200 mm; hypothalamus 300 mm; and hippocampus 350 mm. [file GLIA-70-89-s006.tif]

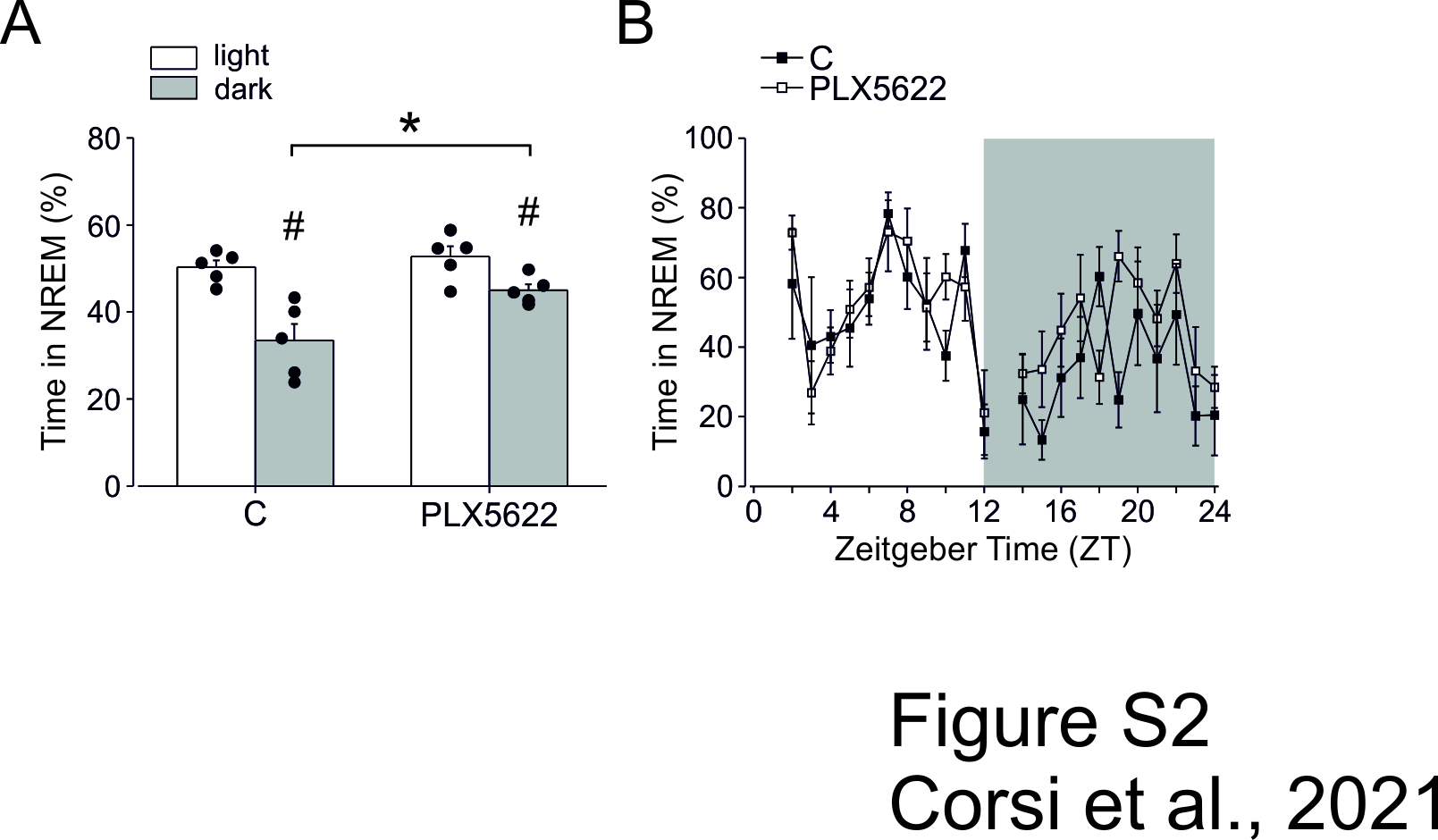

Supplement: Supplementary file 3 — Figure S2 Effect of microglial depletion on NREM sleep duration in the dark and light phases. A. Mean ± s.e.m. and individual distribution of NREM sleep duration in 22 h of light vs 22 h of dark in C57BL/6N (C) and PLX5622‐treated mice. (C light: 50.27 ± 1.57%, C dark: 33.45 ± 3.79%, n = 5, p = 0.02 corrected, z = 2.00, t = 0.00; PLX5622 light: 52.73 ± 2.37%, PLX5622 dark: 44.96 ± 1.41%, n = 5, p = 0.02 corrected, z = 2.00, t = 0.00, Wilcoxon test; light: C vs PLX5622, p = 0.26, z = 0.6, U = 9.0; dark: C vs PLX5622, p = 0.018 corrected, z = −2.1, U = 2.0, Mann–Whitney test). B. Time‐courses of NREM sleep. Data are expressed as a percentage of the total time analyzed and are shown as mean ± s.e.m. [file GLIA-70-89-s004.tif]

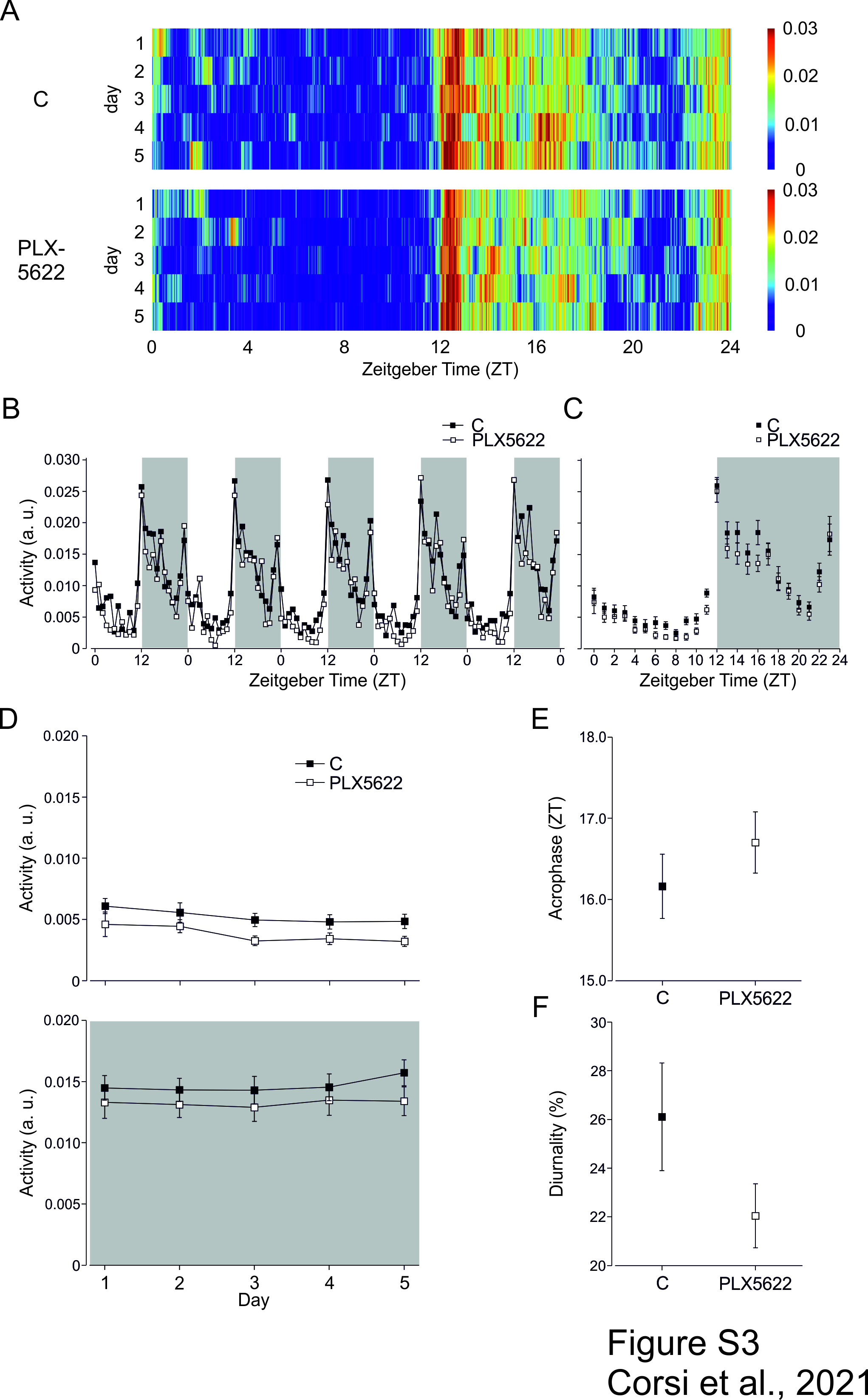

Supplement: Supplementary file 4 — Figure S3 Motor activity and circadian variables recorded with DVC in light–dark condition. A. Color‐coded heatmaps for control (C, top) and PLX5622 group (bottom) showing the raw activity recorded by DVC for 5 days under light–dark condition. B. Average of hourly activity over 5 days in control (C, black squares, n = 18) and PLX5622 (white squares, n = 15); s.e.m. are omitted for graphical clarity. C. Hourly activity averaged over 5 days for control (C,) and PLX5622‐treated mice. D. Five days time course analysis of activity along 12 h of light (top) and 12 h of dark (bottom). No significant difference was observed between the two groups in light and dark condition (p > 0.05, Two‐way ANOVA for repeated measures). E. Mean of the acrophase (C: 16.16 ± 0.39 CT; PLX5622 16.70 ± 0.37 CT, p = 0.33, Student's t‐test). F. diurnality for control (C) and PLX5622 groups (C: 26.10 ± 2.21%; PLX5622 22.04 ± 1.30%, p = 0.14, Student's t‐test). Data are shown as mean ± s.e.m. [file GLIA-70-89-s002.tif]

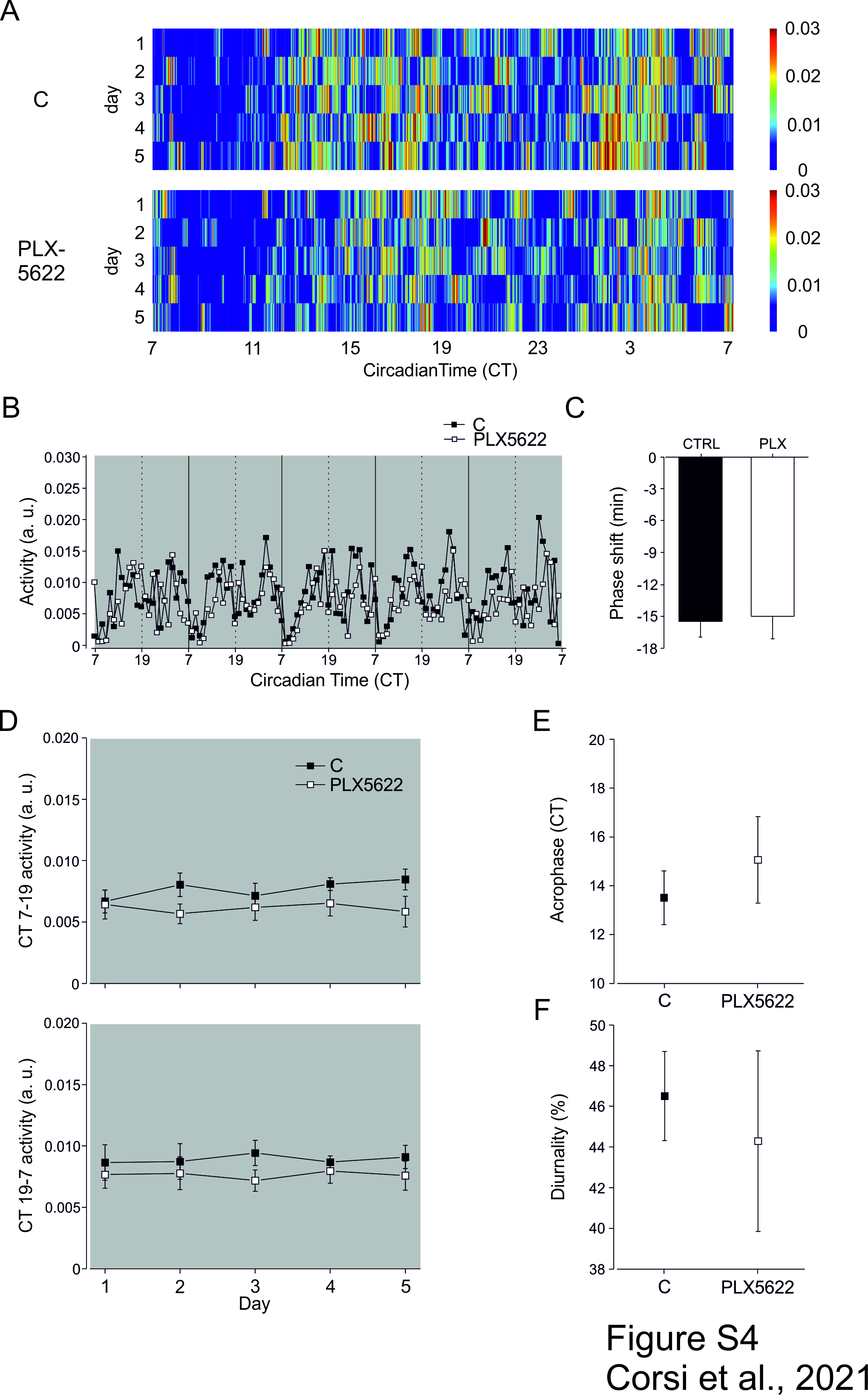

Supplement: Supplementary file 5 — Figure S4 Motor activity and circadian variables recorded with DVC in dark–dark condition. A. Color‐coded heatmaps for control (C, top) and PLX5622 group (bottom) showing the raw activity recorded by DVC for 5 days of total darkness. B. Average of hourly activity over 5 days in control (C, black squares, n = 6) and PLX5622 (white squares, n = 5); s.e.m. are omitted for graphical clarity. C. Phase shift for control (C) and PLX5622‐treated mice in constant darkness (C: −15.46 ± 1.51 CT; PLX5622–14.99 ± 2.10 CT p = 0.85, Student's t‐test, days 8–26). D. Time course analysis of activity in constant darkness along CT 7–19 (top) and CT 19–7 (bottom). No significant difference was observed between the two groups in light and dark condition (p > 0.05, Two‐way ANOVA for repeated measures) E. Mean of the acrophase (C: 13.50 ± 1.10 CT; PLX5622 15.06 ± 1.77 CT, p = 0.46, Student's t‐test) and F. diurnality for control (C) and PLX5622 groups (C: 46.50 ± 2.19%; PLX5622 44.28 ± 4.44%, p = 0.64, Student's t‐test). Data are shown as mean ± s.e.m. [file GLIA-70-89-s007.tif]

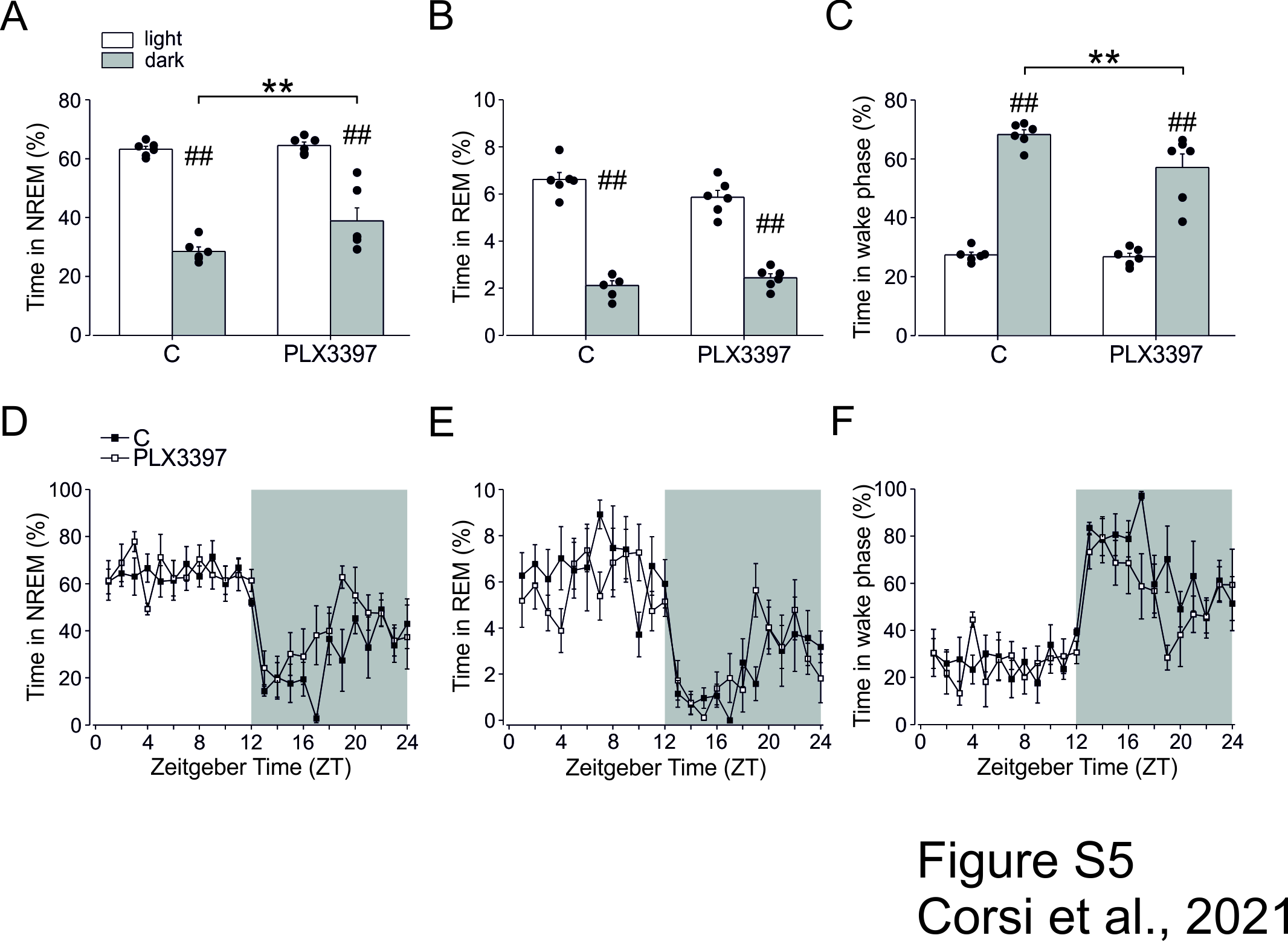

Supplement: Supplementary file 6 — Figure S5 Effect of microglial depletion with PLX3397 on sleep and wake duration in the dark and light phases. A‐C. Time spent in NREM (A), REM (B) and Wake (C) during the light and the dark period by the mice before (C) and after microglial depletion (PLX3397). NREM: light C 63.23 ± 0.96, dark C 28.51 ± 1.5%, light PLX3397 64.49 ± 1.11%, dark PLX3397 38.89 ± 4.34%. REM: light C 6.61 ± 0.29, dark C 2.12 ± 0.2%, light PLX3397 5.85 ± 0.30%, dark PLX3397 2.43 ± 0.17%; WAKE: light C 27.36 ± 0.94, dark C 68.23 ± 1.63%, light PLX3397 26.75 ± 1.19%, dark PLX3397 57.04 ± 4.66%. D‐F. Hourly percentage of time spent in NREM (D), REM (E) and wake (F) over 24 hours by the mice before (C) and after microglial depletion (PLX3397). Data are expressed as percentage of the total time analyzed and are shown as mean ± s.e.m. ## p < 0.01 (Wilcoxon test, p < 0.005 = 0.05 corrected); ** p < 0.01, * p = 0.017 (Mann‐Withney test, p < 0.005 = 0.05 corrected). ## p < 0.001 (Two‐way ANOVA, repeated measures, followed by Bonferroni post‐hoc test). [file GLIA-70-89-s003.tif]

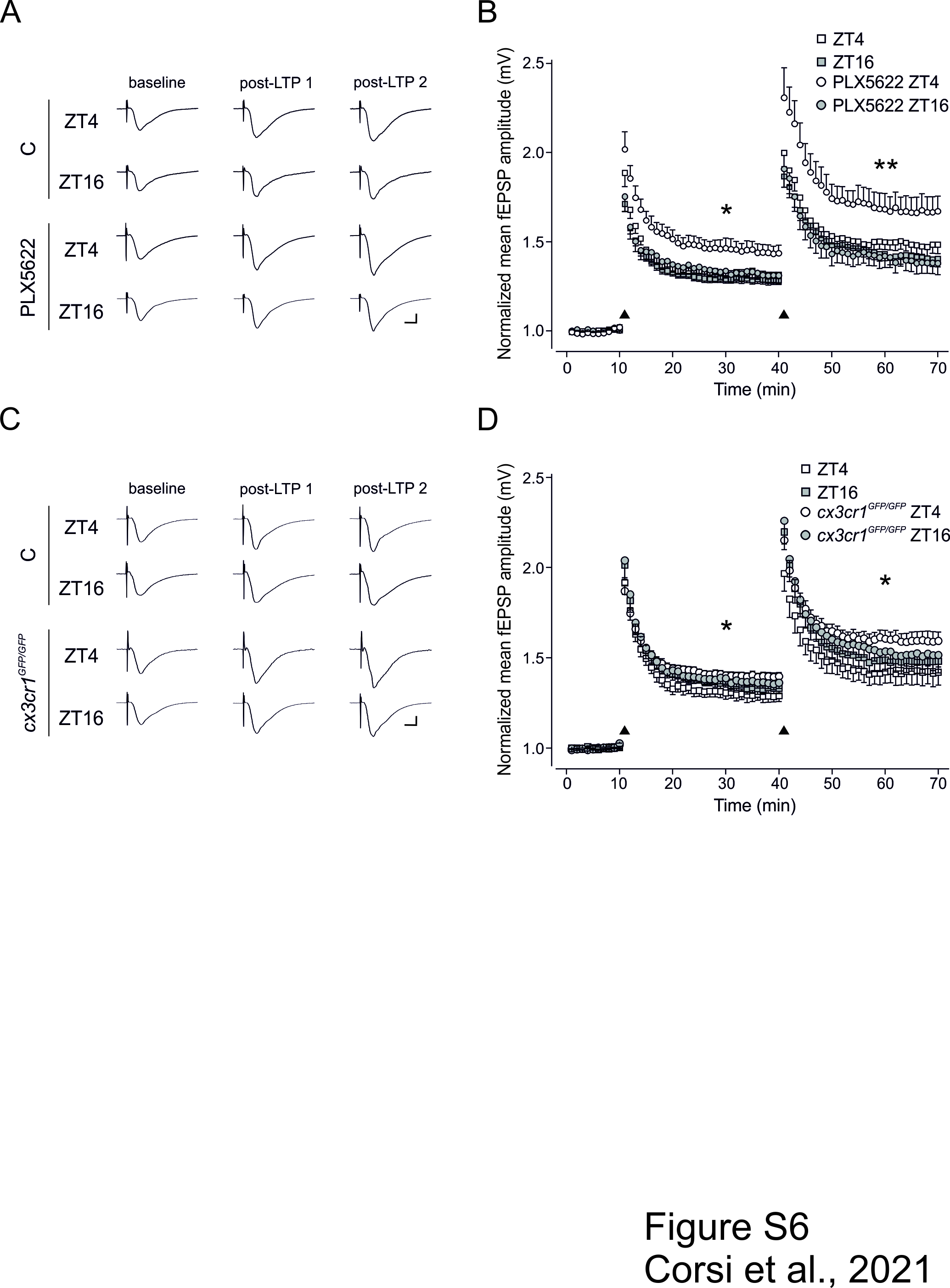

Supplement: Supplementary file 7 — Figure S6 Hippocampal CA1 LTP is affected by PLX5622 and cx3cr1 deletion only in the light phase. A. Representative fEPSP traces for LTP recorded in control (C) and PLX5622 conditions at ZT4 and ZT16 during baseline, at 30 min (post‐LTP 1) and at 60 min (post‐LTP 2); scale bars: 0.3 mV (vertical), 5 ms (horizontal). B. In control conditions (C), LTP amplitude was similar at ZT4 and ZT16 (white and gray squares, respectively) following both the first and second tetanus. PLX5622 treatment causes an increase of LTP amplitude at ZT4 compared to ZT16 (white and gray circles, respectively; * p = 0.016, ** p = 0.005, PLX5622 ZT4 vs ZT16). Arrows indicate time of application of HFS (100 Hz trains of 1 sec duration, 30 min apart). C. Representative fEPSP traces for LTP recorded in control (C) and cx3cr1 GFP/GFP mice at ZT4 and ZT16 during baseline, at 30 min (post‐LTP 1) and at 60 min (post‐LTP 2); scale bars: 0.3 mV (vertical), 5 ms (horizontal). D. In control conditions (C), LTP amplitude was similar at ZT4 and ZT16 (white and gray squares, respectively) following both the first and second tetanus. cx3cr1 deletion causes an increase of LTP amplitude at ZT4 compared to ZT16 (white and gray circles, respectively; first train: * p = 0.037, second train: * p = 0.03, cx3cr1 GFP/GFP ZT4 vs ZT16). Arrows indicate time of application of HFS (100 Hz trains of 1 sec duration, 30 min apart). Data are shown as mean ± s.e.m. [file GLIA-70-89-s005.tif]
